# Investigating the Chemical Profile of Underexplored Parts of *Dipteryx alata* (Baru) Using the PS–MS Technique

**DOI:** 10.3390/plants13131833

**Published:** 2024-07-03

**Authors:** Bruna Vieira Nunes, Viviane Dias Medeiros Silva, Ana Luiza Coeli Cruz Ramos, Talvane Coelho, Angelita Cristine de Melo, Ricardo Manuel de Seixas Boavida Ferreira, Rodinei Augusti, Reinaldo Farias Paiva de Lucena, Júlio Onésio Ferreira Melo, Raquel Linhares Bello de Araújo

**Affiliations:** 1 Departamento de Alimentos, Faculdade de Farmácia, Campus Belo Horizonte, Universidade Federal de Minas Gerais, Belo Horizonte 31270-901, MG, Brazil; brunavieiranuunes@gmail.com (B.V.N.); analuizacoeli@gmail.com (A.L.C.C.R.); raquel@bromatologiaufmg.com.br (R.L.B.d.A.); 2 Departamento de Ciências Exatas e Biológicas, Campus Sete Lagoas, Universidade Federal de São João del-Rei, Sete Lagoas 36307-352, MG, Brazil; vivianedms05@gmail.com (V.D.M.S.); coelhotalvane@gmail.com (T.C.); 3 Curso de Farmácia, Campus Centro-Oeste, Universidade Federal de São João del-Rei, Sete Lagoas 36307-352, MG, Brazil; angelitamelo@ufsj.edu.br; 4 Instituto Superior de Agronomia, Universidade de Lisboa, 1649-004 Lisboa, Portugal; rbferreira@isa.ulisboa.pt; 5 Departamento de Química, Campus Belo Horizonte, Universidade Federal de Minas Gerais, Belo Horizonte 31270-901, MG, Brazil; augusti.rodinei@gmail.com; 6 Instituto de Biociências, Universidade Federal de Mato Grosso do Sul, Cidade Universitária, Campo Grande 79070-900, MS, Brazil; reinaldo.lucena@ufms.br

**Keywords:** fingerprints, *Fabaceae*, baru, Cerrado

## Abstract

The baru (*Dipteryx alata* Vog.), a fruit native to the Cerrado biome, is well-known for its almonds, which are extensively exploited and exported. Unfortunately, the remaining parts of this fruit are often discarded. This study investigates the fixed chemical constituents of the baru, including the bark, pulp, endocarp, and almonds, using the PS–MS technique in positive and negative ionization modes. Notably, this research presents the first chemical profile of baru almonds in both their raw and roasted states. The analysis identified 57 compounds reported for the first time in a baru and 24 common compounds. The majority of these compounds are classified as flavonoids. In both ionization modes, the peel exhibited a higher proportion of phenolic compounds, although the chemical compounds varied among the peel, pulp, almond, and endocarp. These findings highlight the perspective of bioeconomy and biotechnology. By staggering baru fruit production alongside extractivists, we can optimize the utilization of all parts of the fruit. Furthermore, given the knowledge of the biological properties of flavonoids and the baru composition, we recommend additional studies to analyze their potential in preventing chronic non-communicable diseases.

## 1. Introduction

The Cerrado accounts for 5% of the planet’s biodiversity and is considered one of the most biodiverse savanna biomes in the world. This biome represents the second-largest vegetation in Brazil [1] and is considered one of the world’s hotspots. This biome is distributed mainly in the country’s central region and occupies approximately 57% of the state of Minas Gerais. Despite the great diversity of endemic species and their relevance in the socio-biodiversity context, this biome is under high pressure and risk of threat [1,2].

Due to its extension and geographical distribution, the Cerrado biome exhibits significant heterogeneity in its natural resources, with emphasis on the abundance of native fruit species [3]. These Cerrado fruits possess high nutritional values and distinct sensory attributes, which hint at a substantial technological potential for food product development within the industry. Moreover, native foods also play a crucial role in the regional context, contributing to both economic and social vitality through extractivism [1].

Among the fruits of the Cerrado, the barueiro (*Dipteryx alata* Vogel) stands out as an arboreal species belonging to the Fabaceae family. Regionally, it is known by various other names, including pau-cumbaru, fruta-de-macaco, cumbaru, cumarurana, barujo and coco-feijão [4]. This versatile tree finds application in a variety of contexts, including food, logging, medicinal, industrial, and landscape use, as well as recovering degraded areas.

**Figure 1:**
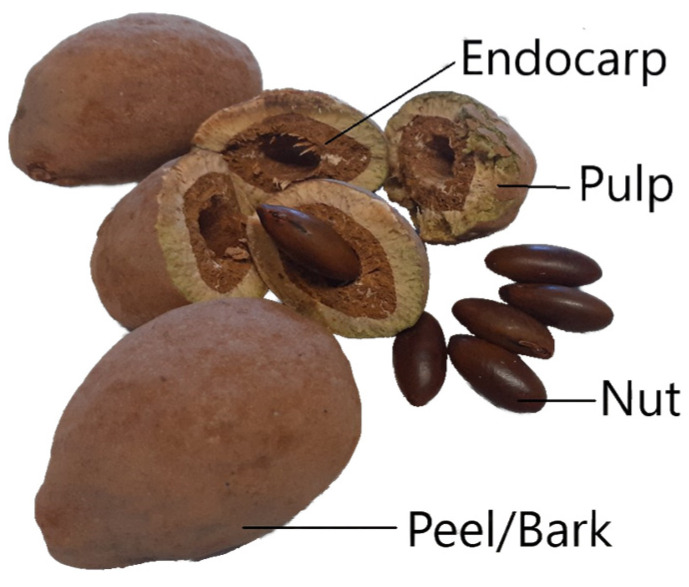
Parts of the baru (*Dipteryx alata* Vog.) fruit.

The barueiro has excellent adaptability, resulting in high productivity with fruits and seeds of excellent quality [5]. In terms of yield, each plant produces between 2000 and 5000 fruits, but not all trees bear fruit annually [6,7]. The fruit, known as baru, is a drupe-ovoid, slightly flattened, and measures approximately 4–5 cm in length. It features a leathery brown epicarp, a fibrous and sweet mesocarp, and a woody endocarp that encloses a single edible seed of about 2–2.5 cm (Figure 1) [6,8].

The baru can assist in treating diseases such as cholesterol, diabetes, gastritis, osteoporosis, sexual impotence, oxidative stress, and metabolic and cardiovascular diseases [9,10,11,12]. This fruit comprises 30% pulp, 5% almond, and 65% endocarp [13], and both the pulp and the almond are edible. Unfortunately, the almonds are the mostly used part, and the remaining parts are often discarded. Additionally, there are several reports in the literature about the physicochemical and nutritional characteristics of baru almonds [14,15,16,17,18,19], while for the pulp, there is less information [7,20,21]

Therefore, investigating the chemical profile of this fruit from the Cerrado biome holds relevance for a deeper understanding of its nutritional value. Such insights can contribute not only to the quality and economic value of the newly developed products but also to sustainable development and biodiversity conservation within the biome. Additionally, they facilitate the integration of other parts of the baru tree into both national and international markets.

There are several forms of chemical characterization, among them the ambient ionization mass spectrometry by paper spray (PS–MS). This technique enables a detailed analysis, including the identification of multiple substances present in complex matrices, making it possible to obtain the fingerprint of the sample in wide mass ranges [2,22]. PS–MS has become popular due to the simplicity and speed of implementation and the possibility of performing in situ analysis [23]. Furthermore, studies have demonstrated that the technique allows rapid analyses with relevant results [24,25]. PS–MS has been used for the analysis of several native Brazilian fruits, such as araticum [2,26] and pequi [25].

Given that baru is an integral part of Brazilian biodiversity and possesses significant nutritional and bioactive characteristics, expanding consumption possibilities is a potentially advantageous endeavor. It could serve as both a food source and an economic alternative for the population. In light of these considerations, this study aims to investigate the fixed chemical constituents of the peel, pulp, endocarp, and almond of baru (*Dipteryx alata* Vog) using the paper spray mass spectrometry (PS–MS) technique in the positive and negative ionization modes.

## 2. Results and Discussion

The baru parts were analyzed using PS–MS in both negative and positive ionization modes. As a result, 65 chemical compounds were tentatively identified across the diverse parts of the baru, as shown in Table 1. The Supplementary Material includes full scan spectra obtained for all baru parts (Figures S1–S10) in both ionization modes. Additionally, the mass spectrum of product ions is presented in Figures S11–S30 (negative mode) and in Figures S31–S40 (positive mode). Among these compounds, 55 were detected in the negative mode. Notably, only nine compounds among the total identified have previously been reported for the baru tree. These include citric acid, ellagic acid, protocatechuic acid hexoside, isoferulic acid, luteolin, vicenin 2, trigalloylglucose, tetragalloylglucose, and pentagalloylglucose.

The presence of chemical compounds varies across the shell, pulp, almond, and endocarp in both ionization modes. In the pulp, 24 compounds were identified in the negative mode, while only four were identified in the positive mode. Interestingly, the total number of compounds identified in the pulp was higher than that reported by Leite et al. [27], who identified 22 compounds in the baru tree. These compounds belong to classes such as polyphenols, flavonoids, terpenes, and fatty acids. Notably, in our study, only four compounds coincided with those identified in the baru parts after ionization in the negative mode: citric acid (*m/z* 191), luteolin (*m/z* 285), protocatechuic acid hexoside (*m/z* 315) and vicenin 2 (*m/z* 593).

The pulp of the baru exhibits a soft, thick, sweet, and astringent composition. It primarily consists of starch, fiber, and sugars and is rich in vitamins and minerals, including potassium, copper, iron, calcium, phosphorus, magnesium, and tannins [7,16,28]. Several studies have evaluated the use of baru pulp in food matrices. Ferreira, Florizo, and Argondoña [21] explored the use of baru pulp flour in biscuit formulation, assessing its stability. Remarkably, no microbial growth was observed during 80 days of storage, rendering the product microbiologically stable. Antunes et al. [7] substituted wheat flour with pulp baru flour to create noodles. These noodles exhibited a high dietary fiber content and lower carbohydrate levels compared to the control sample (which used 100% wheat flour). Silva et al. [8] successfully developed a fermented alcoholic beverage using baru pulp. The results aligned with current Brazilian legislation for fermented fruits, providing a new source of use for this raw material that has been explored little.

Baru almonds, typically the most sought-after part of the fruit, boast a pleasant taste and find widespread acceptance for culinary purposes. Beyond their palatability, they stand out for their high nutrient density, high market value, and their role as part of an abundant genetic heritage [29]. According to the literature, fresh baru almonds exhibit the following average composition: 37.13% carbohydrates, 31.73% lipids, 22.96% proteins, 14.44% total fibers, 6.63% water, and 1.55% ashes. Furthermore, baru almonds contain unsaturated fatty acids (mainly oleic and linoleic acids), minerals (boron, zinc, copper, manganese, and magnesium), antioxidant molecules such as polyphenols (catechin, caffeine, rutin, gallic acid, chlorogenic acid, o-coumaric, and trans-cinnamic acid) and vitamins C and E (alpha and gamma tocopherols) [18,19,30]. Baru almonds are classified as a good-quality protein source, with an amino acid score value corrected by protein digestibility of approximately 90% [17]. Consequently, its consumption is recommended for humans as a complementary protein or a substitution for animal protein [11].

This study represents the first investigation into the chemical profile of baru almonds, both in natural and roasted forms. This analysis provides insights into the distinctive compounds that may be associated with the heat from roasting. We tentatively identified 29 compounds in fresh almonds and 31 in roasted almonds. Among these, 24 are common for both. Thus, seven are present only in roasted almonds, while five are found exclusively in fresh almonds. In the natural state of baru almonds, eight compounds were tentatively identified in the positive ionization mode and 19 in the negative mode, a value similar to that found by Oliveira-Alves et al. [15] in the roasted almonds, where 20 compounds were detected in the negative mode. Among these, five compounds were tentatively identified in the present study (isoferulic acid, ellagic acid, trigalloylglucose, tetragalloylglucose, and pentagalloylglucose).

The consumption of almonds typically happens after roasting, and they can be used in various culinary preparations. Silva-Luis et al. [9] concluded in their study that baru seed oil reduced platelet aggregation and lowered the production of reactive oxygen species. Additionally, it improved vascular function, suggesting its potential as a functional oil for the prevention and treatment of cardiovascular diseases. According to Campidelli et al. [14], hyperlipidemic diets supplemented with baru almonds or baru almond paste resulted in reduced levels of total cholesterol and a lower risk of cardiovascular disease. The authors concluded that a high-fat diet supplemented with baru almonds and baru almond paste promoted metabolic benefits in rats, which may suggest similar beneficial effects in humans. In fact, almond oil is used in folk medicine to combat high fever, as a menstrual regulator, and to treat rheumatism [27].

Despite the significant number of compounds identified in the baru almonds, 82.4% of the phenolics identified were present in the peel of this fruit for the negative ionization mode. Fruit peels typically exhibit a high total phenolic content since these compounds are secondary metabolites of plants, which protect fruits against ultraviolet light, pathogens, parasites, and predators [11]. Interestingly, only one study was found in the literature exploring the potential of baru peel flour as an alternative in the enrichment and formulation of foods [8].

Furthermore, the endocarp, which constitutes 65% of the total volume of the baru fruit, contains more chemical compounds than both the pulp and almond. In both ionization modes, a total of 43 phytoconstituents were identified in the endocarp. According to Rambo et al. [20], the crude bio-oil recovered from the baru endocarp boasts a high hydrocarbon content and contains a fatty acid, cis-vaccenic acid, widely used in the fast-food industry. Additionally, a separate study highlighted its potential as an activated biochar, demonstrating its efficacy as an adsorbent [31].

Table 1 shows that among the classes of phytochemicals, only flavonoids, phenylpropanoids, and benzoic acid derivatives appeared in both ionization modes. In the negative mode, tannins, organic acid, and anthraquinone also occurred. In the positive mode, a steroid was identified. Flavonoids represented 87.5% of the tentatively identified compounds, mainly in the negative ionization mode.

**Table 1:** The chemical profile of the baru in negative mode PS (−) MS and in positive mode PS (+) MS.

Compound	Precursor ion (*m/z*)	Ionization Mode	Fragments (MS/MS)	Parts of the Baru					Reference
				AN	AT	C	E	P	
**Organic acids**									
Citric acid	191	-	191, 111, 87, 85			X		X	[27,32]
**Benzoic acid derivatives**									
Hydroxytrimesic acid	267	-	163, 119			X			[33]
Salicylic acid *O*- glucoside	299	-	137		X	X			[33,34]
Ellagic acid	301	-	301, 229			X	X	X	[15]
Vanillin hexoside	313	-	151			X			[33]
Protocatechuic acid hexoside	315	-	153, 152, 109, 108			X	X		[27,33,34]
Homovanillic acid hexoside	343	-	181			X	X		[33]
Syringic acid hexoside	359	-	197			X	X	X	[33]
Ellagic acid derivative	799	+	395	X	X	X	X	X	[35]
**Steroids**									
Stigmasterol	395	+	269,215	X	X				[36]
**Phenylpropanoids**									
Isoferulic acid	193	-	178, 149, 134		X	X		X	[15]
Hydroxybenzyl-malic acid (eucomic acid)	239	-	195, 179, 177, 133			X			[34]
Dihydrochrysin (pinocembrin)	255	-	135	X	X	X	X		[34]
*p*-*O*-Methylpiscidic acid	269	-	209, 179, 148			X	X		[33]
Fukiic acid	271	-	271, 181, 165, 151, 109			X	X		[33]
Naringenin			153, 135 271, 151						[32]
Butein/Butin			271, 135, 91						
p-Coumaroyl-malic acid	279	-	119		X	X			[33]
Coutaric acid or Phaseolic acid	295	-	135, 133, 115			X			[33]
Feruloyl-malic acid	309	-	193			X	X		[33]
Coumaroylhexose or p-Coumaric acid hexoside	325	-	163, 119	X	X	X	X	X	[32,33]
Phenyllactic acid 2-*O*-hexoside	327	-	165, 147			X	X		[37]
Caffeoylhexose or Caffeic acid-*O*-hexoside	341	-	179	X	X	X	X	X	[33,38]
Ferulic acid hexoside I	355	-	193			X			[33]
Butein-hexoside	433	-	433, 271, 135				X		[32]
Tricaffeoyl-quinic acid	678	+	515, 351			X	X		[35]
**Flavonoids**									
Formonetin	267	-	163, 119			X			[34]
Genistein	269	-	153, 133			X	X		[34]
Trihydroxyl-flavone									[33]
Luteolin	285	-	285, 217, 199, 175, 151, 133			X	X	X	[27,32,33]
Calycosin		+	270, 241, 137	X	X	X			[39]
Eriodictyol	287	-	287, 151, 135			X	X		[32]
Chrysoeriol (diosmetin)	299	-	284		X	X			[33]
Quercetin	301	-	273, 271, 179, 151	X		X	X	X	[33,34]
Myricetin	317	-	287, 179			X			[33]
Apigenin 8-C-glucoside (Isovitexin)	431	-	413, 371, 341, 311, 269		X				[33,34]
Naringenin 6-C-*β*-D-glucoside (hemipholin) ou Naringenin 7- *O*-glucoside (prunin)	433	-	343, 313, 271				X		[34]
Naringenin-*O*-hexoside			433, 271						[32]
Quercetin-arabinofuranoside			301, 300						[33]
Quercetindeoxyhexose	447	-	301, 300			X			[33,34]
Kaempferol-*O*-glucoside			285, 284						[33,34,38]
Orobol/Luteolin-*O*-hexoside;			447, 285, 284, 255						[32]
Isoorientin			429, 387, 357, 327						[33]
Eriodictyol-*O*-glucoside	449	-	449, 287, 269, 259, 151			X			[32]
Quercetin-3-*O*-glucouronide	477	-	301, 179			X			[40]
Dihydromyricetin (ampelopsin) 3′-*O*-*β*-D-glucopyranoside	481	-	463, 355, 193			X		X	[34]
Noricaritin hexoside	533	-	371			X		X	[33]
Isoschaftoside	563	-	503, 473, 443, 353, 383		X	X		X	[34]
(-)-Theaflavin			545, 519, 425						
Kaempferol 3-*O*-*α*-L-arabinopyranosyl-7-*O*-*α*-*L*-rhamno pyranoside			431, 285						
Phloretinxyloglucoside	567	-	435				X		[40]
Apigenin 6,8-di-C-glucoside (vicenin 2)	593	-	503, 383	X	X	X	X		[27,33,34,37]
Naringenin di-C-hexoside	595	-	475, 449, 385, 355, 329	X	X	X	X		[34]
Eriodictyol-*O*-hexose-*O*-rhamnose or Eriodictyol-7-*O*-rutinoside (eriocitrin)			595, 459, 433, 287						[32,37]
Phloretin-3′,5′-di-C-glucoside	597	-	597, 477, 429, 417, 399, 315	X	X	X	X	X	[32,37]
Myrecitin-3-*O*-(2″-*O*-galloyl)-pentoside	601	-	449	X	X	X			[40]
Quercetin hexose deoxyhexose	609	-	489, 463, 447, 301			X	X	X	[33,34]
Kaempferol di-hexoside			447, 285						[33,38]
Isorhamnetin 3-*O*-rutinoside			477, 315						[38]
Hesperidin (Hesperetin-*O*-rutinoside)			609, 301						[32]
Isorhamnetin3-*O*-(2″-*α*-arabinopyranosyl)-*β*-glucopyranoside	623	-	477		X		X	X	[38]
Myricetin hexose deoxyhexose	625	-	463, 317, 316	X			X	X	[34]
Myrecitin-3-*O*-(2″-*O*-galloyl)-hexoside	631	-	479, 317				X		[40]
Quercetin-acetyl-rutinoside	651	-	609	X	X	X	X		[33]
Luteolin-7-*O*-hexosyl-8-C-(6″-acetyl)-hexoside			489, 327						[40]
Isorhamnetin-*O*-rhamnosylarabinoside-O-glucoside	725	-	417	X	X	X	X	X	[38]
Quercetin-rhamnosylacetyl-hexoside-rhamnoside	797	-	651	X	X	X	X	X	[33]
Schoepfin A derivative of (iso)mangiferin	839	-	839, 821, 749, 331	X	X		X	X	[37]
Schoepfin A derivative of (iso)mangiferin	841	+	661, 559, 541, 523, 509, 491, 475, 439, 423	X	X		X		[37]
Nothofagin derivative of (iso)mangiferin	855	-	855, 735	X	X	X	X	X	[37]
Nothofagin derivative of (iso)mangiferin	857	+	677, 659, 641, 599, 575, 557, 509, 487, 439, 369, 357, 327				X		[37]
Astragaloside I	870	+	671, 455	X	X	X	X	X	[39]
Aspalathin derivative of (iso)mangiferin	871	-	871	X	X	X	X	X	[37]
	873	+	819, 807, 731, 675, 658, 631, 616, 604, 591, 573, 561, 489, 459, 447, 387, 369, 357, 303, 289	X	X		X	X	[37]
**Tannins**									
Trigalloylglucose	635	-	331	X		X		X	[15]
Tetragalloylglucose	787	-	787	X	X	X	X		[15]
Tetrahydroxyxanthone-C-hexoside	841	-	841, 823, 805, 559, 329	X		X	X	X	[37]
Procyanidin trimer	850	-	697	X	X	X	X	X	[40]
Pentagalloylglucose	939	-	939, 635	X	X		X	X	[15]
**Others**									
Medicarpin	269	-	254, 210			X	X		[34]
4,10-Dihydroxy-3,9-dimethoxypterocarpan	317	+	280	X	X			X	[39]
Sutherlandin	741	+	303	X			X		[41]

AN: natural almond; AT: toasted almond; C: peel; E: endocarp; P: pulp; X: identified.

All ions tentatively identified for the positive mode are presented for the first time. Regarding the baru fruit, only pulp and toasted almonds were the targets of the studies presented. This means that all the other parts presented here in this study (natural almond, peel, and endocarp) are being studied for the first time for the chemical profile.

According to Oliveira-Alves et al. [15], trigalloylglucose (*m/z* 635), tetragalloylglucose (*m/z* 787), and pentagalloylglucose (*m/z* 939) are gallotannins (GTs). These compounds consist of a central molecule, such as glucose, surrounded by gallic acid units (GA). For instance, pentagaloylglucose comprises five gallic acid units bound to glucose. GTs are important phenolic compounds found in walnuts. Their high antioxidant potential arises from the extensive hydroxylation of aromatic rings. These authors showed that baru seed extracts showed an antiproliferative effect on HT29 cells (models used for in vitro cancer studies), probably due to the presence of GTs and GA. These three TGs were tentatively identified in most of the parts of the baru analyzed in the present study.

GTs are polyphenols that belong to the class of hydrolyzable tannins and are present in fruits such as raspberries, blackberries, strawberries, walnuts, grapes, and pomegranates. Their reported biological activities span a wide range, including reduced incidence of cardiovascular disease, diabetes, cataracts, inflammation, and inhibition of tumor growth. Studies have shown that GTs inhibit the proliferation of various tumor cells, including those associated with colorectal and prostate cancer, without exerting toxicity to normal cells [15].

According to Leite et al. [27], citric acid (*m/z* 191), found mainly in citrus fruits, has chelating and buffering characteristics, preventing food browning and prolonging shelf life. This is the first time this compound has been reported in baru peel. These authors also report that flavonoids, such as luteolin, present in the pulp of the fruited *D. alata*, may decrease the intracellular production of free radicals. This compound (*m/z* 285) was tentatively identified in the peel and endocarp of the baru.

A study by Lima et al. [10] presents several promising findings regarding the health benefits of consuming baru. These include its potential to treat metabolic diseases, reduce oxidative stress, combat cancer atherogenesis, and address microbial infection. The unsaturated carbohydrates found in baru exert an anti-inflammatory effect on the cardiovascular system, helping to reduce blood cholesterol concentrations. Baru also plays a relevant anti-inflammatory role in the prevention of skin aging and tissue protection against oxidative stress [42].

Furthermore, the ion *m/z* 841, a symmetrical dimer of mangiferin, was identified in all baru parts studied. It was isolated from the peel of the stem of the hose *Cyclopia genistoides* and exhibited moderate antiviral activity [37]. Salem et al. [38] isolated several compounds in *Astragalus sieberi* (Fabaceae) using LCI–ESI–MS. These compounds demonstrated effective cytotoxic activity against colon and breast carcinoma cell lines, suggesting their potential as antitumor agents. Additionally, studies on other species within the same family as baru have associated the presence of flavonoids from *Astragalus* with antitumor activities [43,44].

Given the chemical profile obtained from the baru tree, it can be emphasized that it is rich in phenolic compounds and their glycosylated forms. Abu-Reidah et al. [33] identified about 100 compounds, most of which are flavonoids and derivatives. They suggested that this profile may partially explain the higher antioxidant activity of broad beans (*Vicia faba* L.) compared to other vegetables and legumes. The authors also encourage the use of broad beans as a source of functional ingredients to develop value-added products that improve health. The same can be extrapolated to the parts of the baru.

Biological investigations have reported different anti-inflammatory mechanisms of eriodyctiol, apigenin, kaempferol, and naringenin and their glycosides. These glycosylated compounds are also tentatively identified in baru. The qualitative characterization of the chemical constituents of the leaf extract of *Gleditsia capsica* (Fabaceae) using UPLC–ESI–MS–MS led to the conclusion that the extract has bioactive potential to be incorporated into topical anti-inflammatory drugs due to its significant flavonoid content [36].

Recent studies have shown that flavonoids perform several essential functions, such as acting as antioxidants, anti-inflammatory agents, antihypertensive agents, and antidiabetic agents. Flavonoids are phenolic compounds of plant origin and have several biological properties, including antioxidant, anti-inflammatory, antibacterial, antiallergic, and vasodilator action effects. These compounds aid in preventing various chronic non-communicable diseases, such as cardiovascular pathologies, oxidative stress, some cancers, atherosclerosis, diabetes, Alzheimer’s, cataracts, and other respiratory disorders. They are concentrated in different parts of the plant [45]. As previously reported, this class of compounds was predominant in the baru, with the majority present in the shell, using the negative ionization mode.

Among these flavonoids, first reported for baru, quercetin and its derivatives exhibit antioxidant, anticarcinogenic, anti-inflammatory, antiaggregant, and vasodilator effects. Additionally, naringenin, which has anti-inflammatory actions, also affects the metabolism of sex hormones, including binding to estrogen receptors [46]. Several other flavonoids, such as anthocyanidins, chalcones, and flavones, serve as plant pigments that determine the color of vegetables [47]. In this study, chalcones including aspalathin, nothofagin, and schoepfin derivatives of (iso)mangiferin, and schoepfin A and nothofagin derivatives of (iso)mangiferin occurred in both ionization modes.

## 3. Materials and Methods

### 3.1. Plant Material

Fresh fruits were collected in Felixlândia (18°45′28″ S, 44°53′56″ W), in the state of Minas Gerais, Brazil, in 2022, at the same harvest and with the same degree of maturity to perform the analysis of the chemical profile. The parts of the fruit (peel, pulp, endocarp, and almond) were separated manually, kept at refrigerator temperature (4 °C), and protected from light in plastic packaging until use. Immediately before the analysis, the almonds were crushed in an analytical mill (IKA A11 Basic, Conshohocken, PA, USA). The roasted almond was acquired in a specialized natural products store in Belo Horizonte, MG, Brazil.

### 3.2. Obtaining the Extracts

To obtain the pulp, peel, endocarp, and almond extracts, 1.0 g of each fresh sample was weighed, previously homogenized, and then 8 mL of methanol was added. The samples were stirred in a vortex for 30 s and kept at rest at room temperature (25 °C) until the analysis of the chemical profile by PS–MS.

### 3.3. Chemical Profile Analysis

The PS–MS analysis of the extracts of the baru parts was conducted in a Thermo LCQ-Fleet mass spectrometer (ThermoScientific, San Jose, CA, USA) in the positive and negative ionization modes. The chromatographic paper was cut with scissors to make triangular papers with a 1.0 × 1.5 × 1.5 cm dimension. The PS source was assembled according to the methodology described by Ramos et al. [26]. The extracts of the samples (2 μL) were applied to the triangular base. After drying, methanol (40 μL) was placed on the paper base, and the tension was applied through the metal clip. The instrumental conditions were as follows: voltage applied to the paper, +4.5 kV (positive mode) and −3.5 kV (negative mode); capillary temperature, 275 °C; capillary voltage, 40 V; and tube lens voltage, 120 V. Full scan mass spectra were acquired in a range of 100–1000 *m/z*. Ionic fragmentation was performed using a collision energy of 15 to 45 units. Data from the mass spectra were processed using Xcalibur software version 2.1 (Thermo Scientific, San Jose, CA, USA). Spreadsheet software (Excel 2020, Microsoft, Redmond, WA, USA) was used to list and organize the average mass spectra for further analysis. The metabolites were supposedly identified by comparing their masses and fragmentation patterns with those described in the literature.

## 4. Conclusions

Baru is composed of various compounds, many with bioactive properties, mainly flavonoids. The ions tentatively identified in this study have not yet been reported for the endocarp and baru peel. These findings can encourage the utilization of these parts, which are often treated as residues, thereby adding value to the fruit and generating income for the local population. Moreover, even for almonds and pulp, which are the most commonly used parts, unpublished data were presented, highlighting the diversity of potential applications of the fruit. In light of these findings, scaling up the production of baru in a sustainable and commercialized manner became feasible, especially within the context of forest bioeconomy and biotechnology. This approach can benefit extractivists and facilitate the insertion of baru production into both national and international markets, promoting a better use of the parts and by-products of the baru.

## Data Availability

Data are contained within the article and its Supplementary Materials.

## Supplementary Materials

The following supporting information can be downloaded at: https://www.mdpi.com/article/10.3390/plants13131833/s1. Figure S1. PS (+) MS of the methanolic extract of the baru pulp; Figure S2. PS (+) MS of the methanolic extract of the baru peel; Figure S3. PS (+) MS of the methanolic extract of the baru endocarp; Figure S4. PS (+) MS of the methanolic extract of the baru seed; Figure S5. PS (+) MS of the methanolic extract of the baru roasted seed; Figure S6. PS (−) MS of the methanolic extract of the baru pulp; Figure S7. PS (−) MS of the methanolic extract of the baru peel; Figure S8. PS (−) MS of the methanolic extract of the baru endocarp; Figure S9. PS (−) MS of the methanolic extract of the baru seed; Figure S10. PS (−) MS of the methanolic extract of the baru roasted seed; Figure S11. Product ion mass spectrum (MS/MS) of the ion of *m/z* 239 (ascribed as deprotonated eucomic acid); Figure S12. Product ion mass spectrum (MS/MS) of the ion of *m/z* 255 (ascribed as deprotonated pinocembrin); Figure S13. Product ion mass spectrum (MS/MS) of the ion of *m/z* 279 (ascribed as deprotonated p-Coumaroyl-malic acid); Figure S14. Product ion mass spectrum (MS/MS) of the ion of *m/z* 287 (ascribed as deprotonated Eriodictyol); Figure S15. Product ion mass spectrum (MS/MS) of the ion of *m/z* 309 (ascribed as deprotonated Feruloyl-malic acid); Figure S16. Product ion mass spectrum (MS/MS) of the ion of *m/z* 313 (ascribed as deprotonated Vanillin hexoside); Figure S17. Product ion mass spectrum (MS/MS) of the ion of *m/z* 317 (ascribed as deprotonated Myricetin); Figure S18. Product ion mass spectrum (MS/MS) of the ion of *m/z* 325 (ascribed as deprotonated Coumaroyl hexose); Figure S19. Product ion mass spectrum (MS/MS) of the ion of *m/z* 341 (ascribed as deprotonated Caffeic acid-O-hexoside); Figure S20. Product ion mass spectrum (MS/MS) of the ion of *m/z* 355 (ascribed as deprotonated Ferulic acid hexoside I); Figure S21. Product ion mass spectrum (MS/MS) of the ion of *m/z* 359 (ascribed as deprotonated Syringic acid hexoside); Figure S22. Product ion mass spectrum (MS/MS) of the ion of *m/z* 431 (ascribed as deprotonated Isovitexin); Figure S23. Product ion mass spectrum (MS/MS) of the ion of *m/z* 449 (ascribed as deprotonated Eriodictyol-O-glucoside); Figure S24. Product ion mass spectrum (MS/MS) of the ion of *m/z* 477 (ascribed as deprotonated Quercetin-3-O-glucouronide); Figure S25. Product ion mass spectrum (MS/MS) of the ion of *m/z* 481 (ascribed as deprotonated Dihydromyricetin (ampelopsin) 3′-O-β-D-glucopyranoside); Figure S26. Product ion mass spectrum (MS/MS) of the ion of *m/z* 533 (ascribed as deprotonated Noricaritin hexoside); Figure S27. Product ion mass spectrum (MS/MS) of the ion of *m/z* 567 (ascribed as deprotonated Phloretinxyl glucoside); Figure S28. Product ion mass spectrum (MS/MS) of the ion of *m/z* 597 (ascribed as deprotonated Phloretin-3′,5′-di-C-glucoside); Figure S29. Product ion mass spectrum (MS/MS) of the ion of *m/z* 623 (ascribed as deprotonated Isorhamnetin3-O-(2″-α-arabinopyranosyl)-β-glucopyranoside); Figure S30. Product ion mass spectrum (MS/MS) of the ion of *m/z* 850 (ascribed as deprotonated Procyanidin trimer); Figure S31. Product ion mass spectrum (MS/MS) of the ion of *m/z* 285 (ascribed as protonated Calycosin); Figure S32. Product ion mass spectrum (MS/MS) of the ion of *m/z* 317 (ascribed as protonated 4,10-Dihydroxy-3,9-dimethoxypterocarpan); Figure S33. Product ion mass spectrum (MS/MS) of the ion of *m/z* 395 (ascribed as protonated Stigmasterol); Figure S34. Product ion mass spectrum (MS/MS) of the ion of *m/z* 678 (ascribed as protonated Tricaffeoyl-quinic acid); Figure S35. Product ion mass spectrum (MS/MS) of the ion of *m/z* 741 (ascribed as protonated Sutherlandin); Figure S36. Product ion mass spectrum (MS/MS) of the ion of *m/z* 799 (ascribed as protonated Ellagic acid derivative); Figure S37. Product ion mass spectrum (MS/MS) of the ion of *m/z* 841 (ascribed as protonated Schoepfin A derivative of (iso)mangiferin); Figure S38. Product ion mass spectrum (MS/MS) of the ion of *m/z* 857 (ascribed as protonated Nothofagin derivative of (iso)mangiferin); Figure S39. Product ion mass spectrum (MS/MS) of the ion of *m/z* 870 (ascribed as protonated Astragaloside I); Figure S40. Product ion mass spectrum (MS/MS) of the ion of *m/z* 873 (ascribed as protonated Aspalathin derivative of (iso)mangiferin).
