# Supplementary material for: Investigating the Chemical Profile of Underexplored Parts of Dipteryx alata (Baru) Using the PS–MS Technique
Source: Plants (Basel). 2024 Jul 3;13(13):1833. doi: 10.3390/plants13131833 (PMC11243931; doi:10.3390/plants13131833)

Figure S1. PS (+) MS of the methanolic extract of the baru pulp.

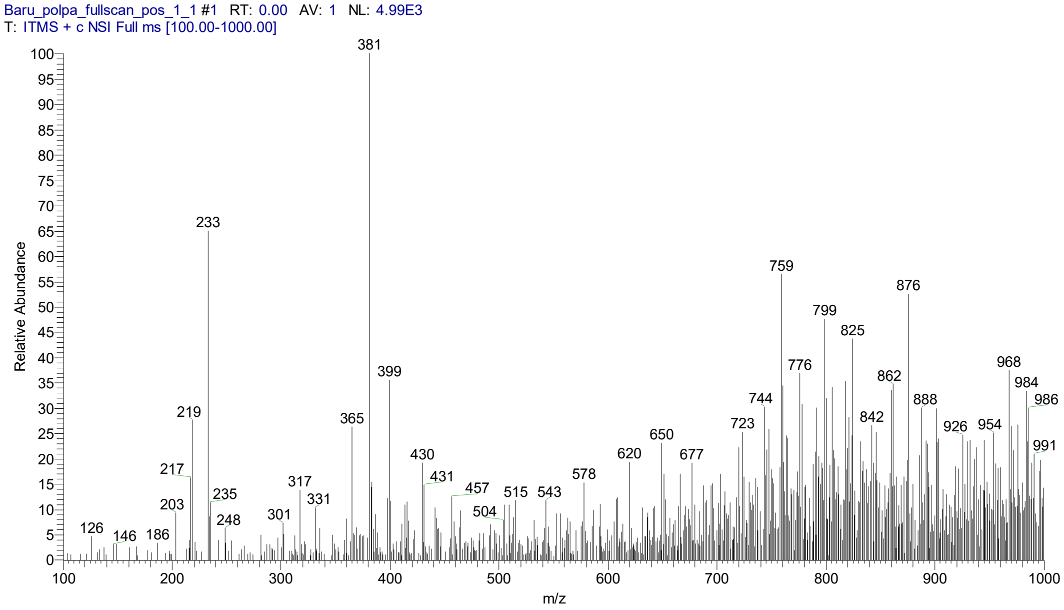

Figure S2. PS (+) MS of the methanolic extract of the baru peel.

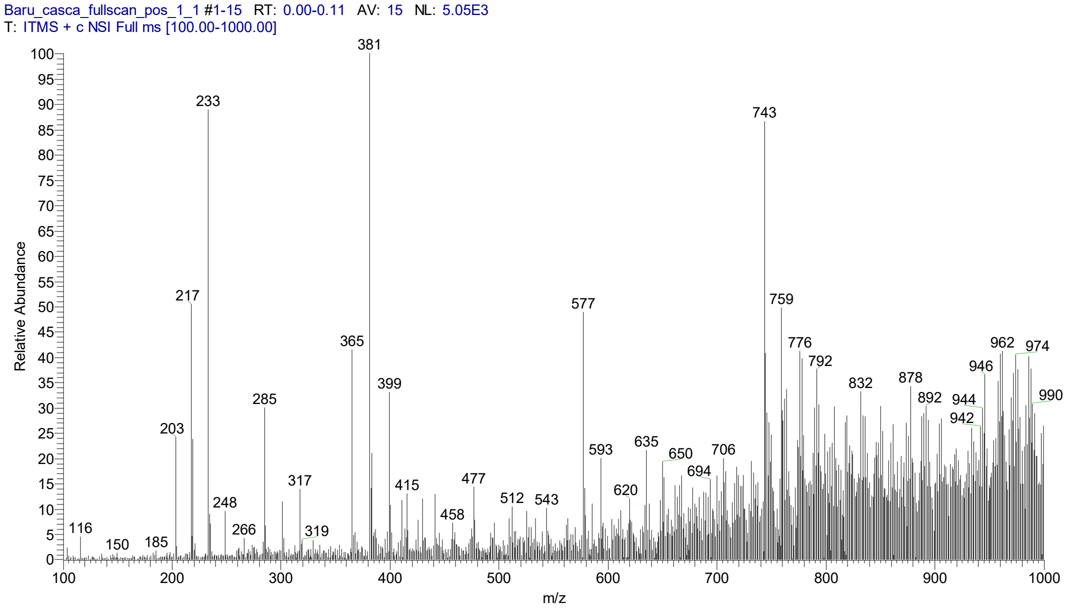

Figure S3. PS (+) MS of the methanolic extract of the baru endocarp.

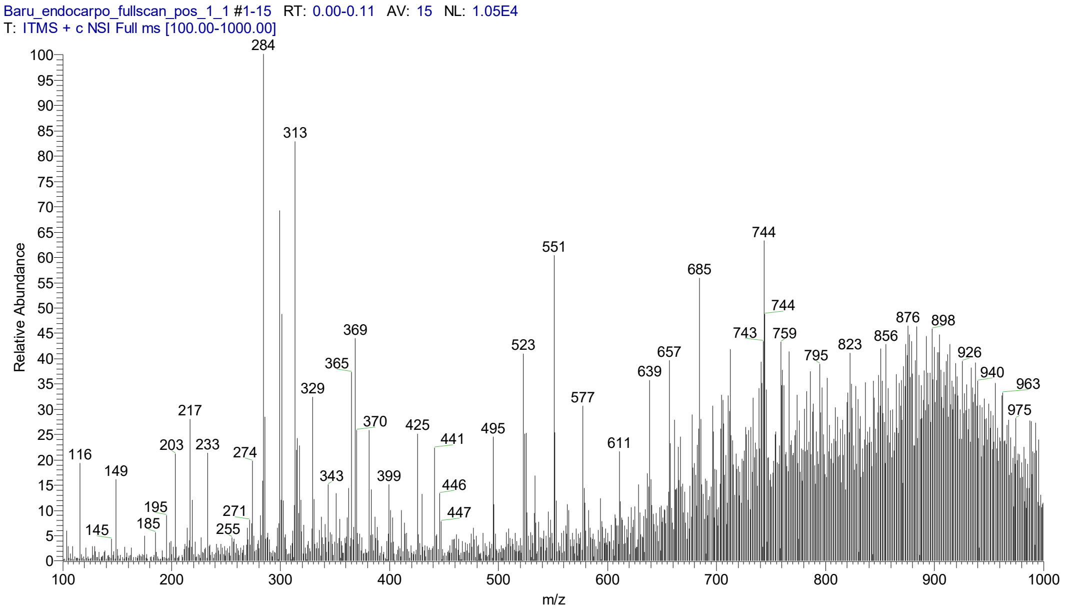

Figure S4. PS (+) MS of the methanolic extract of the baru seed.

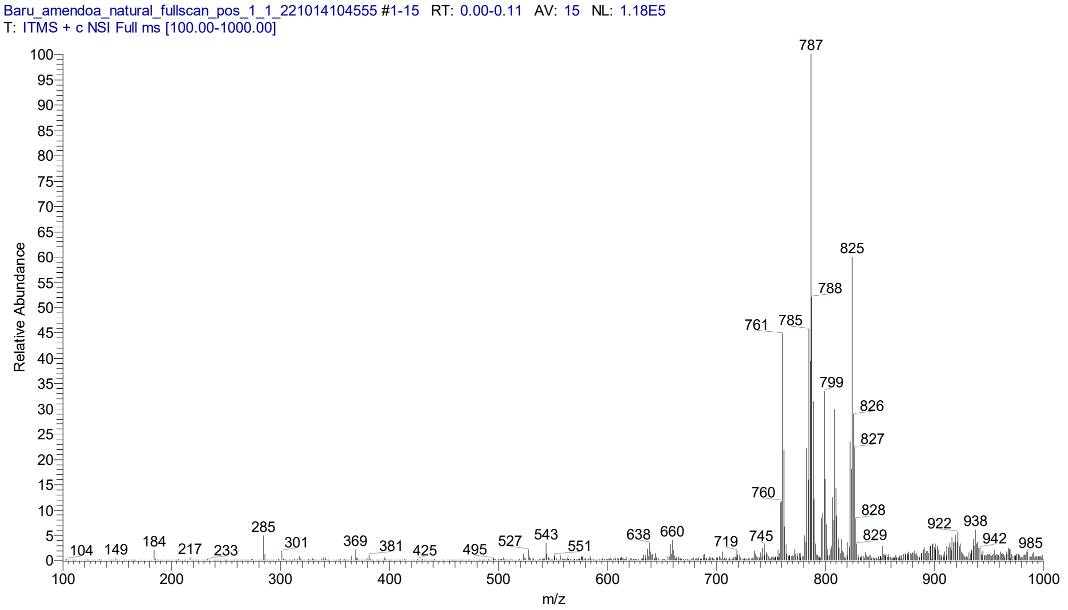

Figure S5. PS (+) MS of the methanolic extract of the baru roasted seed.

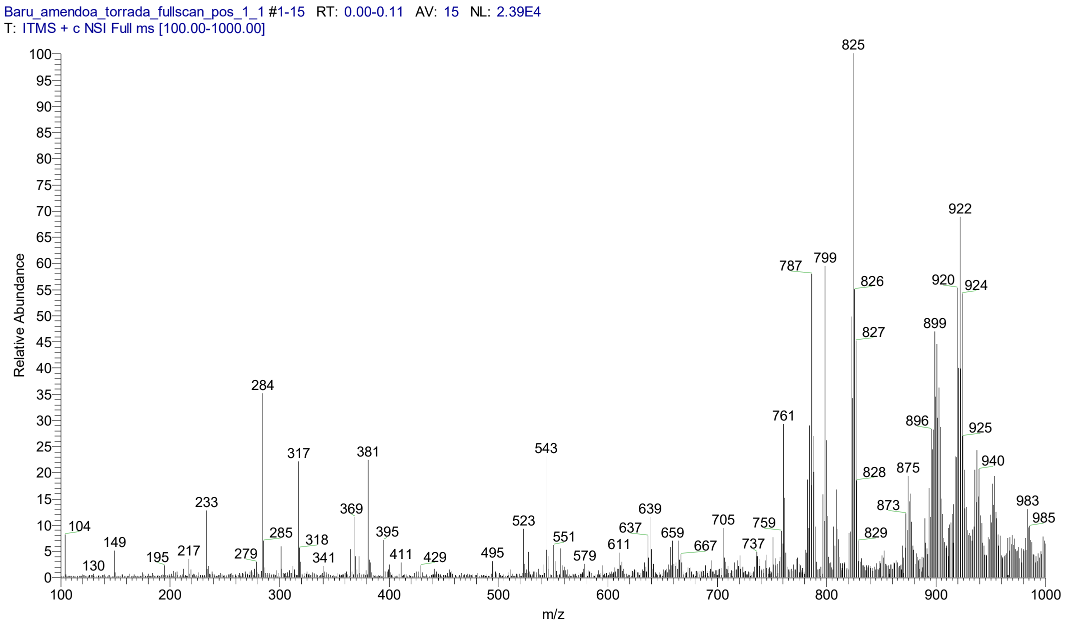

Figure S6. PS (-) MS of the methanolic extract of the baru pulp.

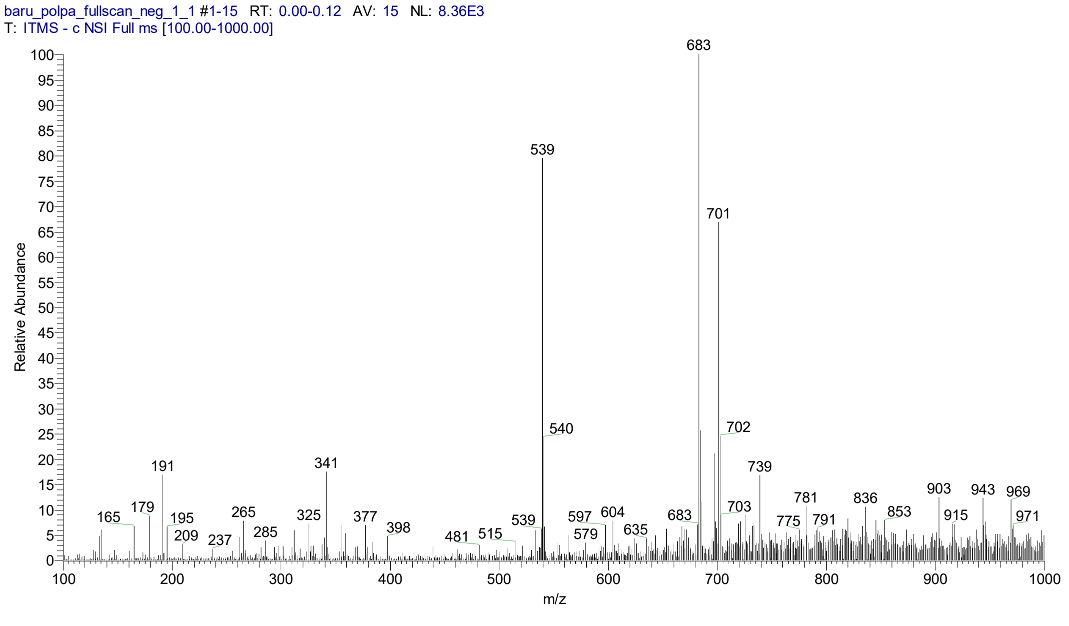

Figure S7. PS (-) MS of the methanolic extract of the baru peel.

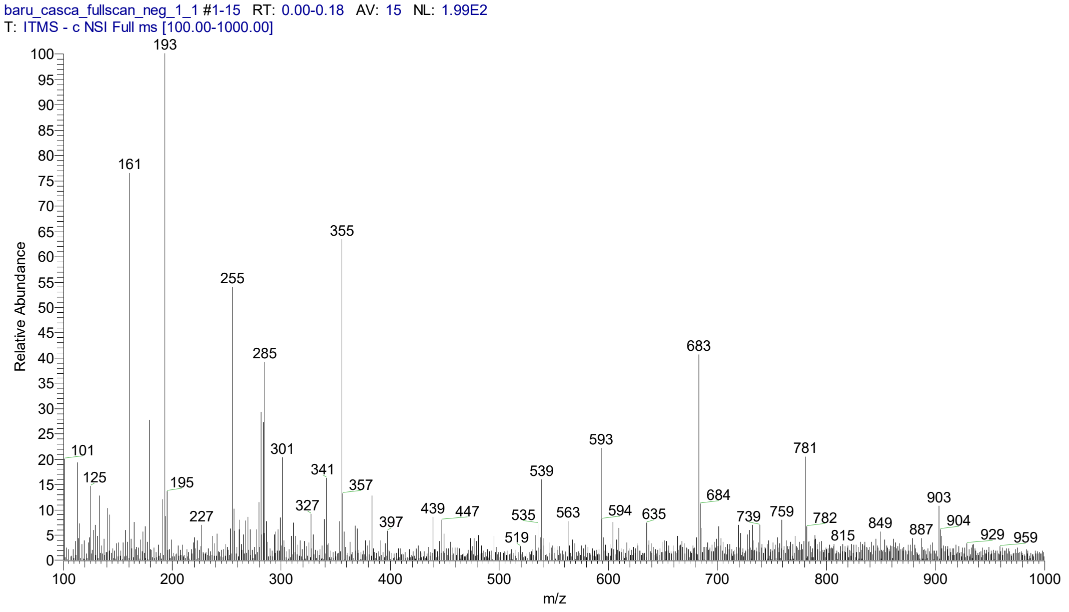

Figure S8. PS (-) MS of the methanolic extract of the baru endocarp.

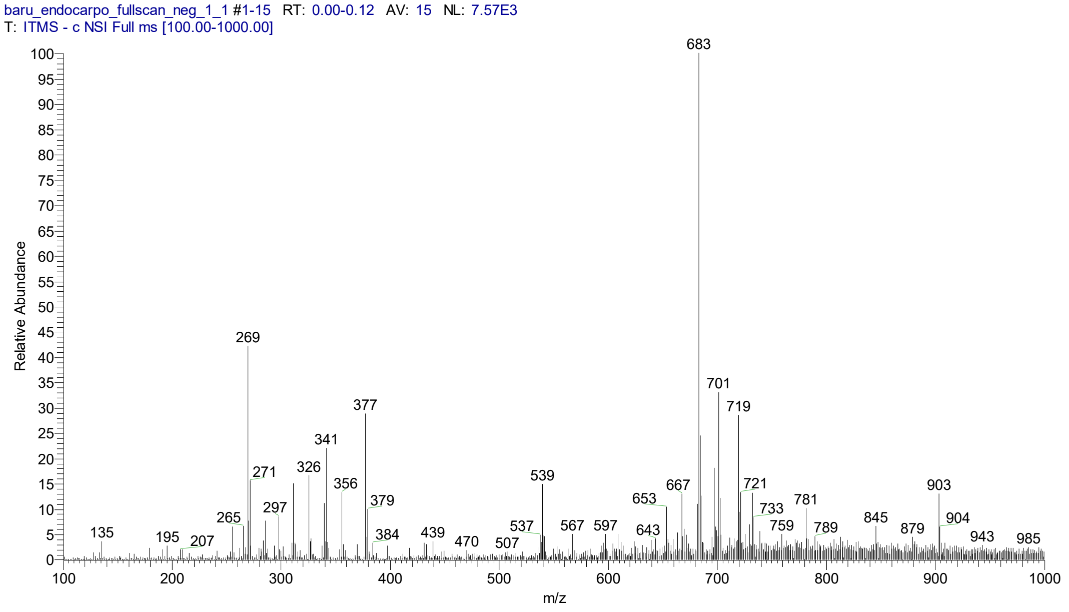

Figure S9. PS (-) MS of the methanolic extract of the baru seed.

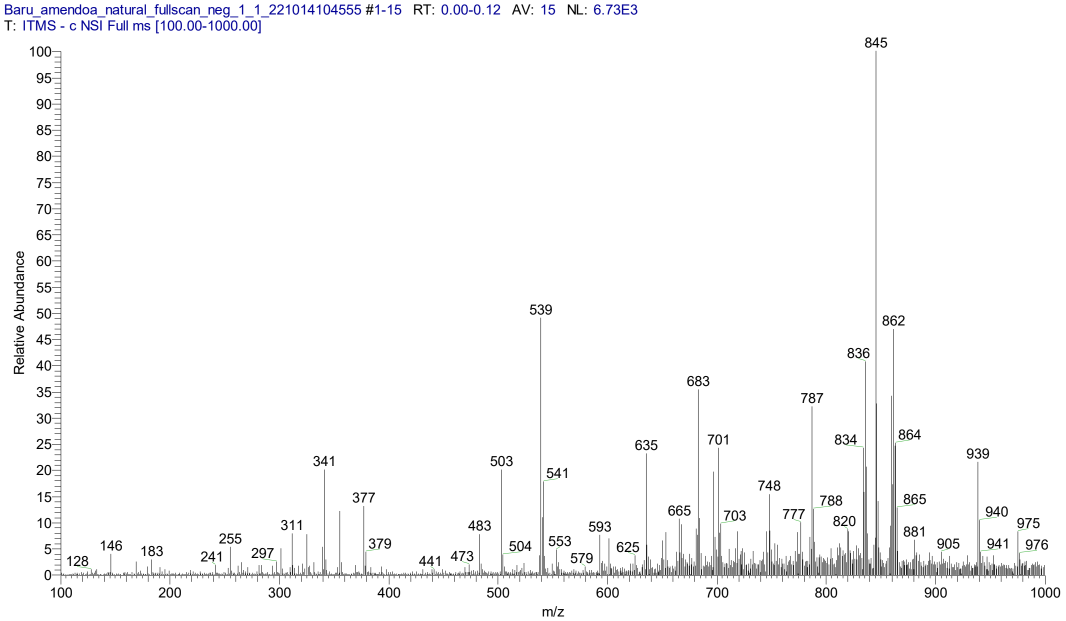

Figure S10. PS (-) MS of the methanolic extract of the baru roasted seed.

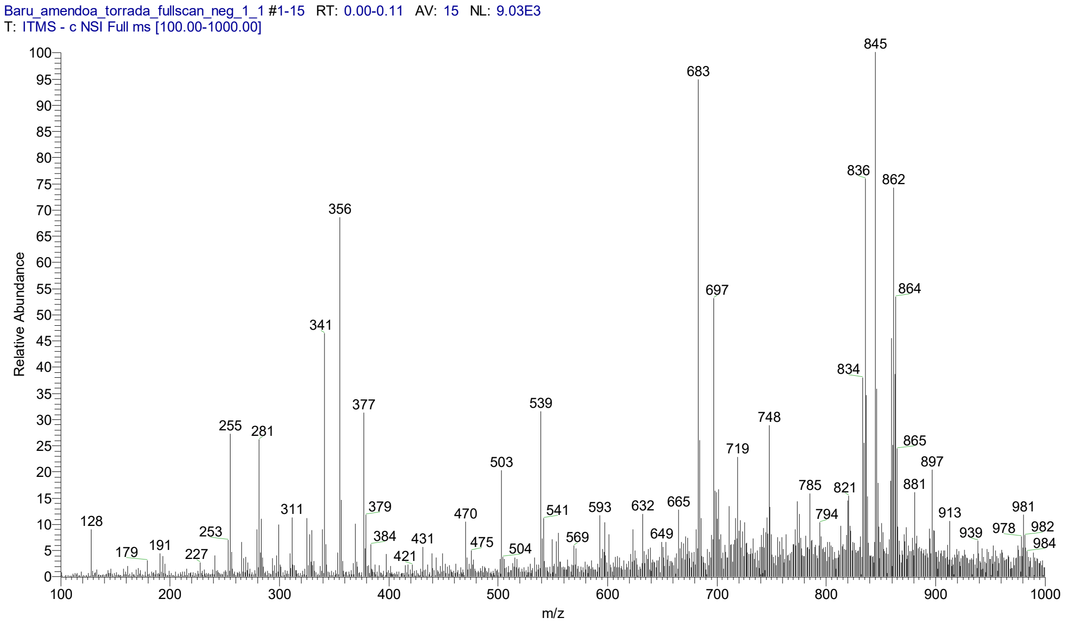

Figure S11. Product ion mass spectrum (MS/MS) of the ion of m/z 239 (ascribed as deprotonated eucomic acid).

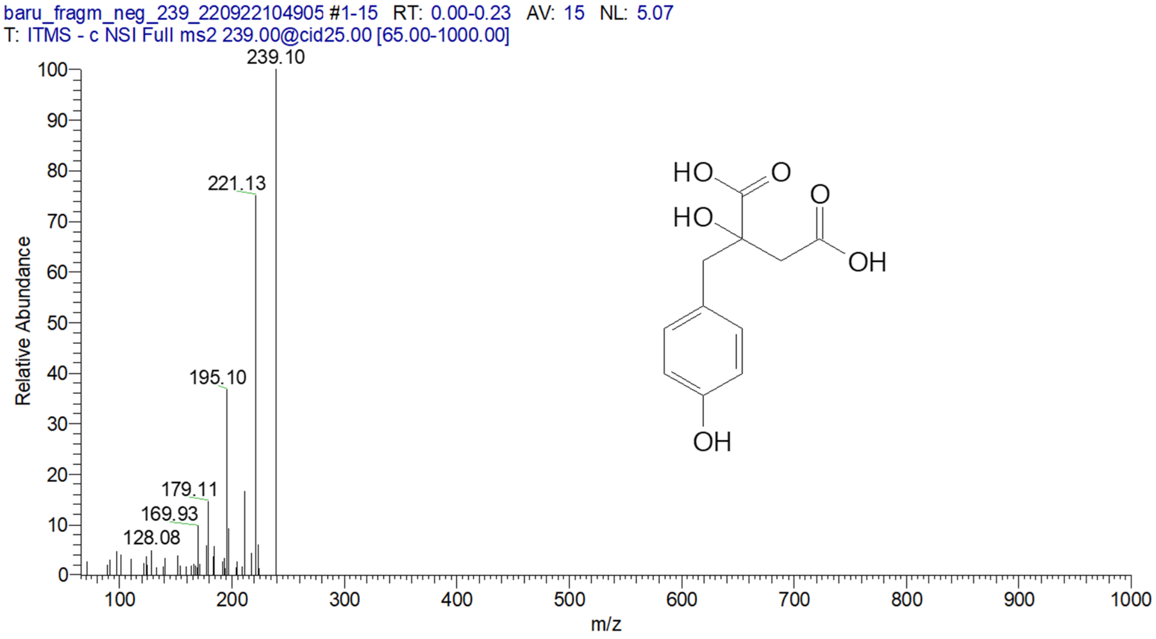

Figure S12. Product ion mass spectrum (MS/MS) of the ion of m/z 255 (ascribed as deprotonated pinocembrin).

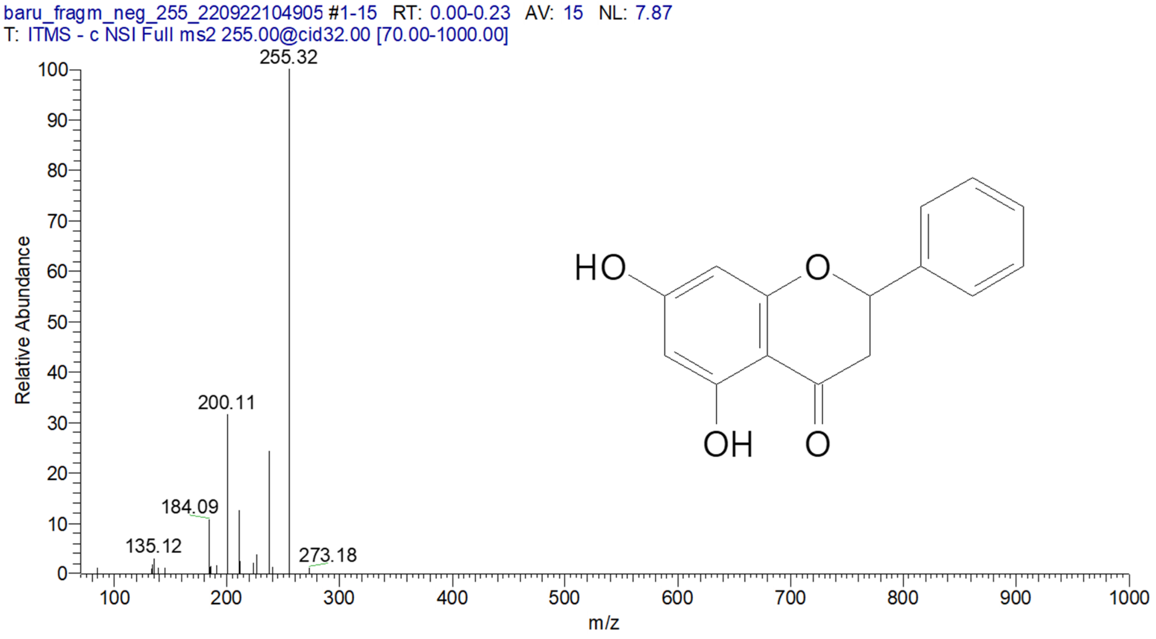

Figure S13. Product ion mass spectrum (MS/MS) of the ion of m/z 279 (ascribed as deprotonated p-Coumaroyl-malic acid).

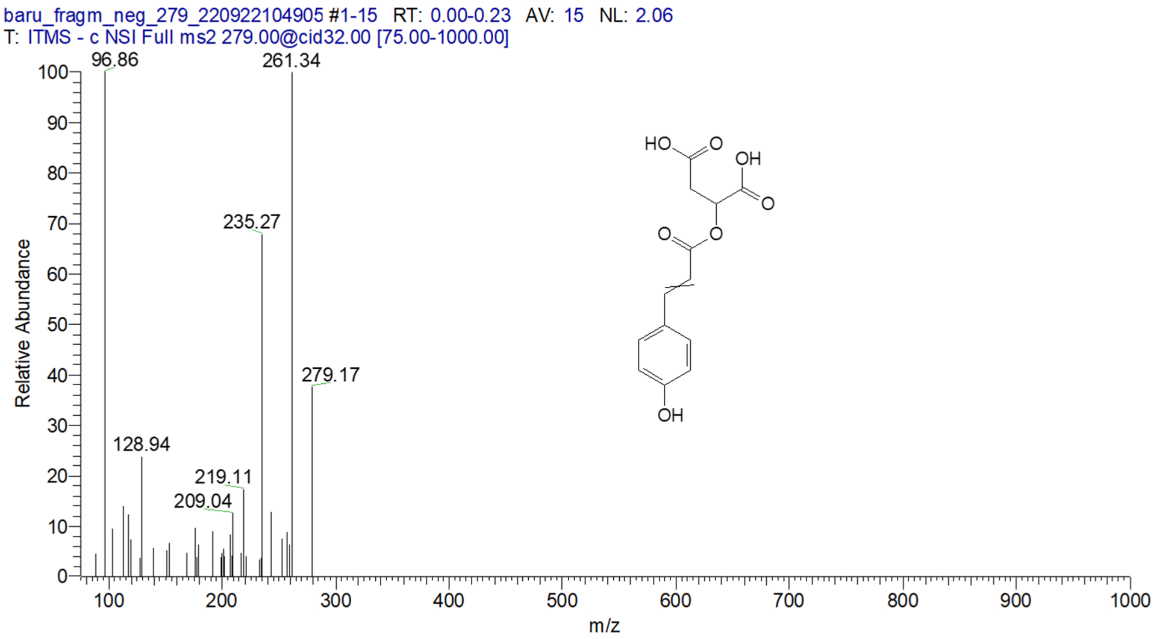

Figure S14. Product ion mass spectrum (MS/MS) of the ion of m/z 287 (ascribed as deprotonated Eriodictyol).

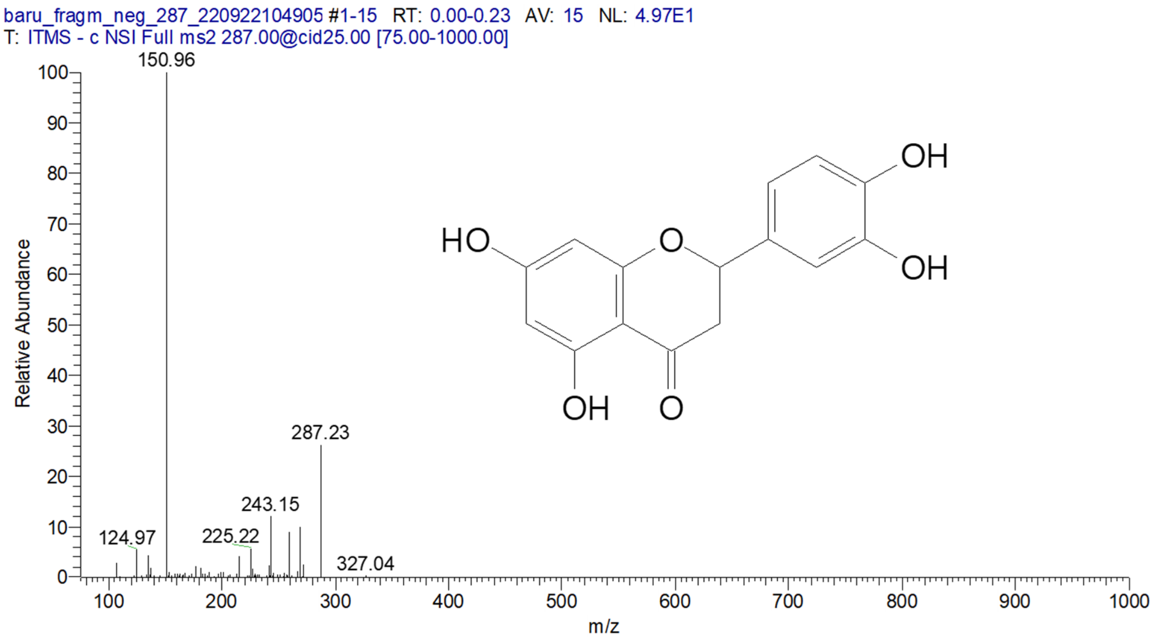

Figure S15. Product ion mass spectrum (MS/MS) of the ion of m/z 309 (ascribed as deprotonated Feruloyl-malic acid).

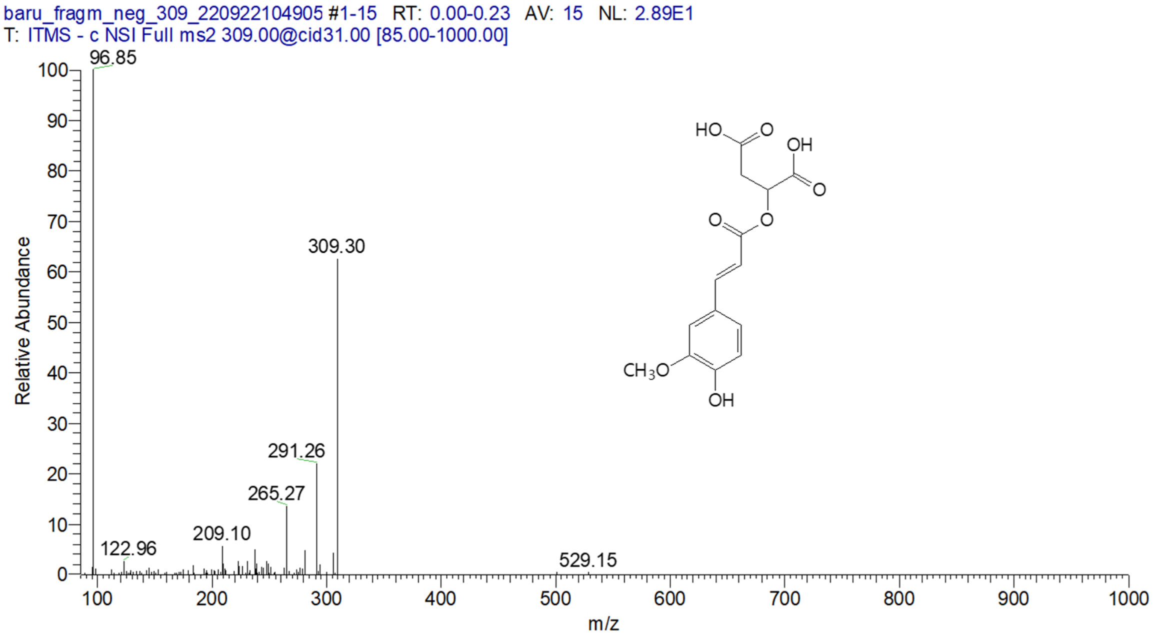

Figure S16. Product ion mass spectrum (MS/MS) of the ion of m/z 313 (ascribed as deprotonated Vanillin hexoside).

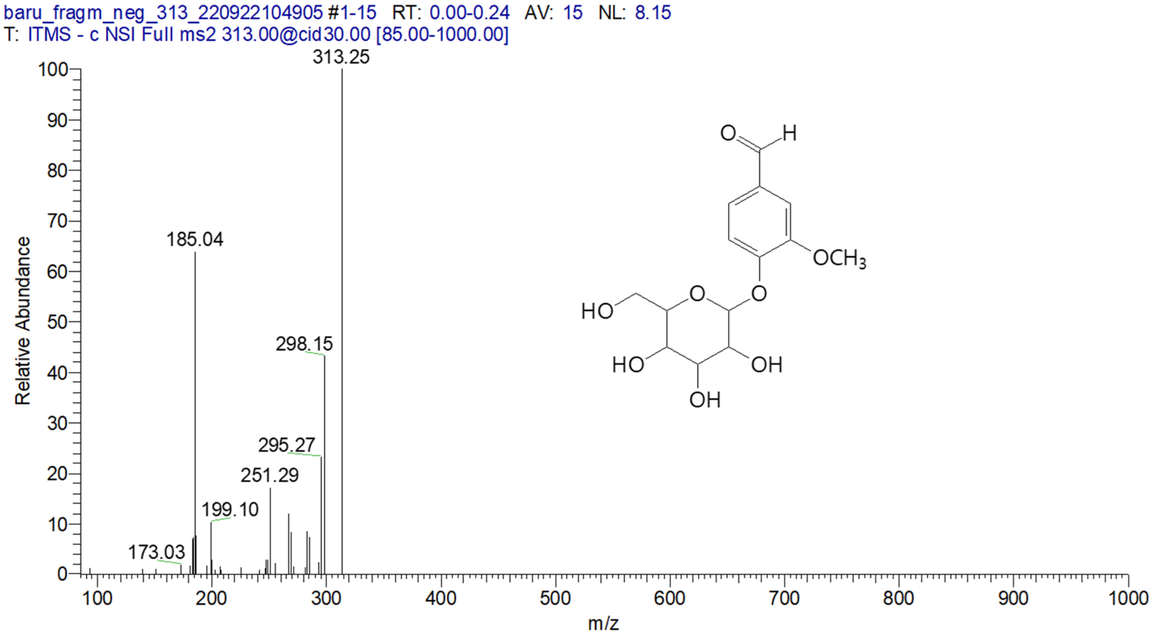

Figure S17. Product ion mass spectrum (MS/MS) of the ion of m/z 317 (ascribed as deprotonated Myricetin).

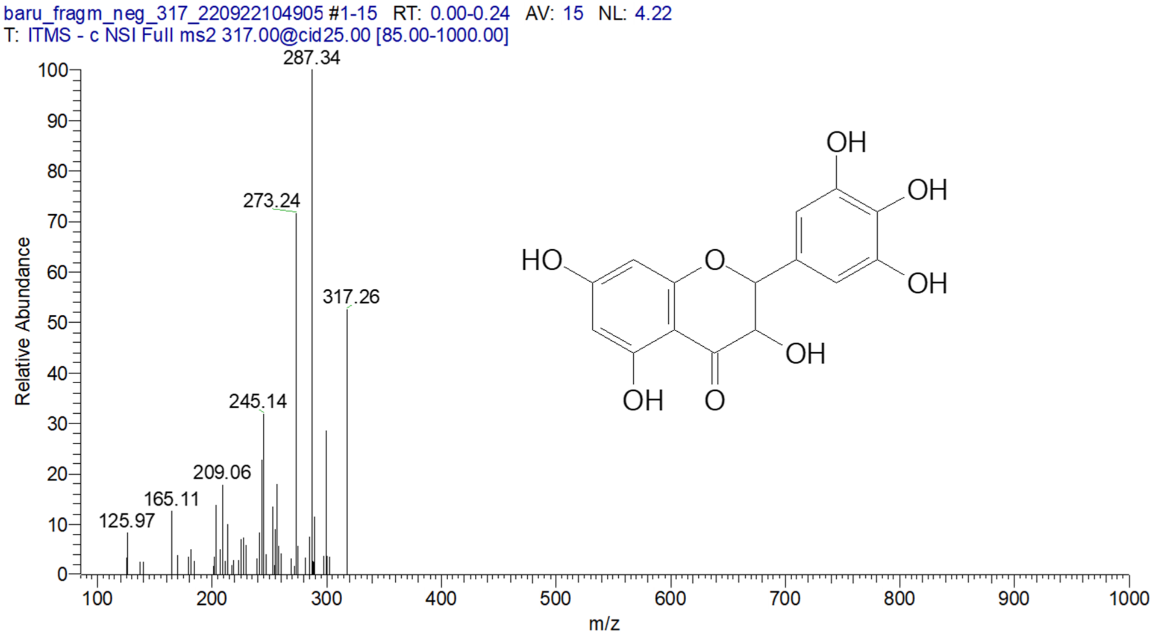

Figure S18. Product ion mass spectrum (MS/MS) of the ion of m/z 325 (ascribed as deprotonated Coumaroyl hexose).

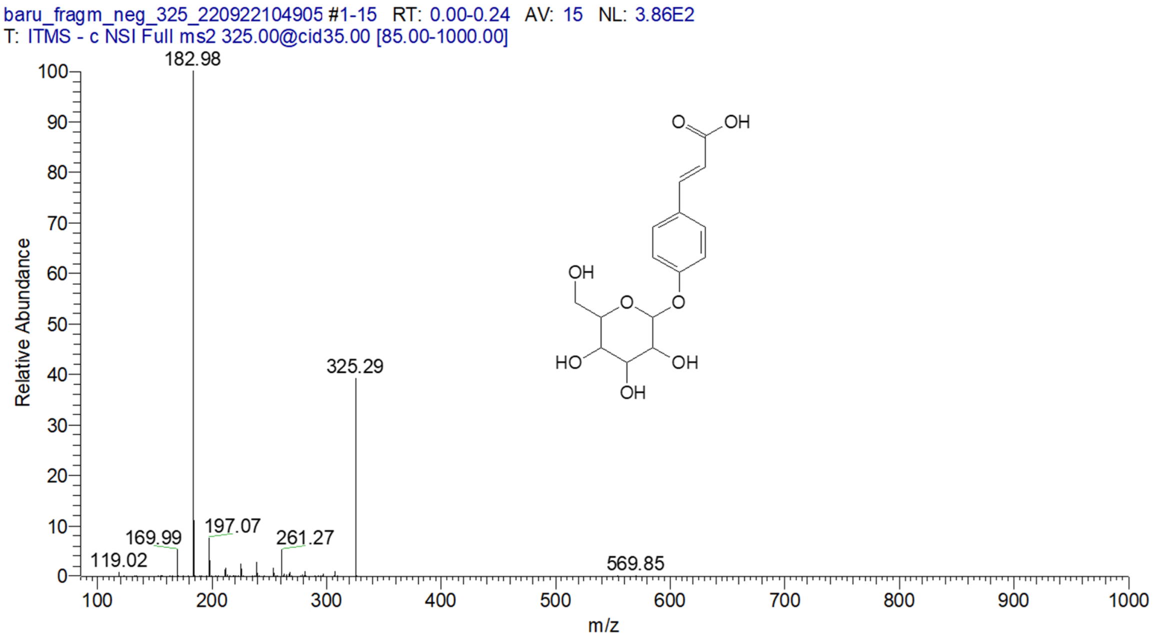

Figure S19. Product ion mass spectrum (MS/MS) of the ion of m/z 341 (ascribed as deprotonated Caffeic acid-O-hexoside).

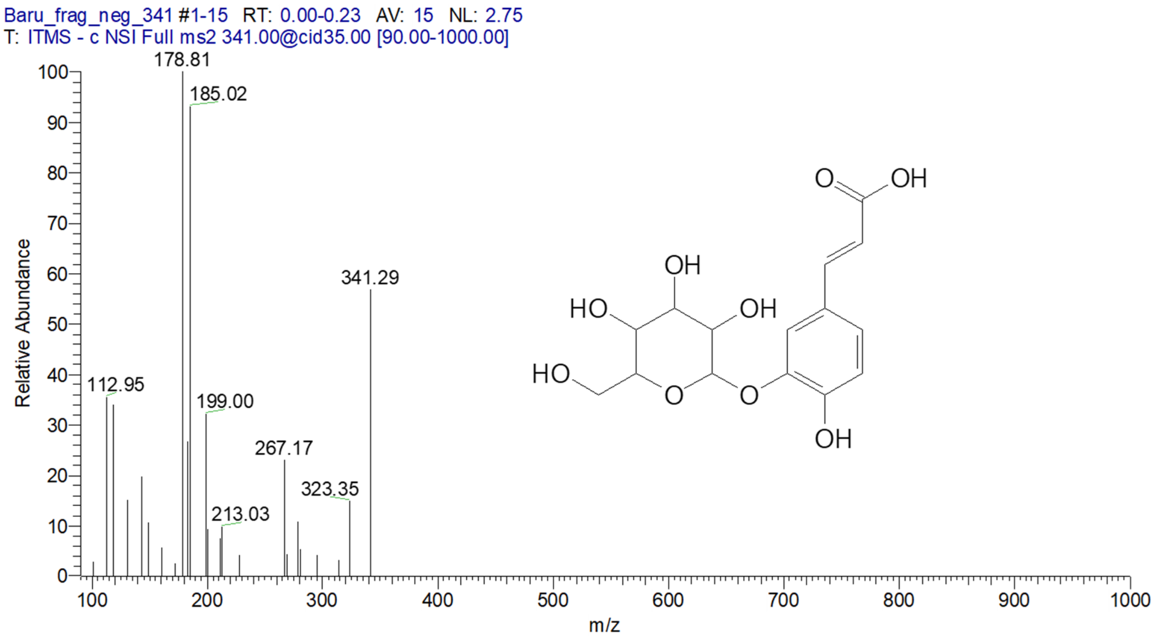

Figure S20. Product ion mass spectrum (MS/MS) of the ion of m/z 355 (ascribed as deprotonated Ferulic acid hexoside I).

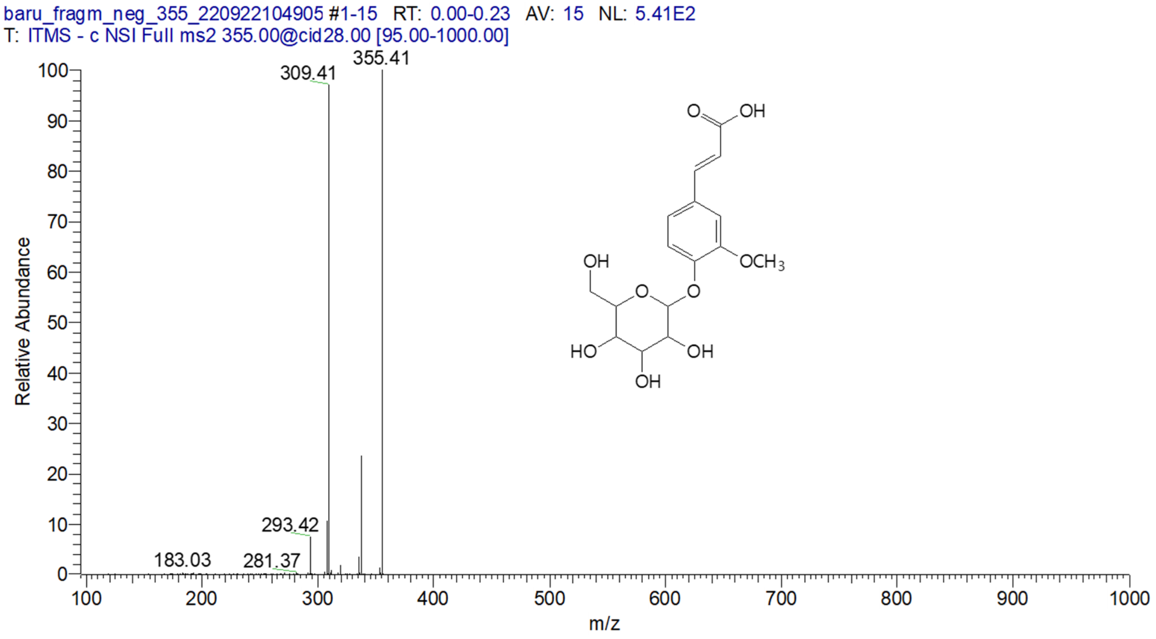

Figure S21. Product ion mass spectrum (MS/MS) of the ion of m/z 359 (ascribed as deprotonated Syringic acid hexoside).

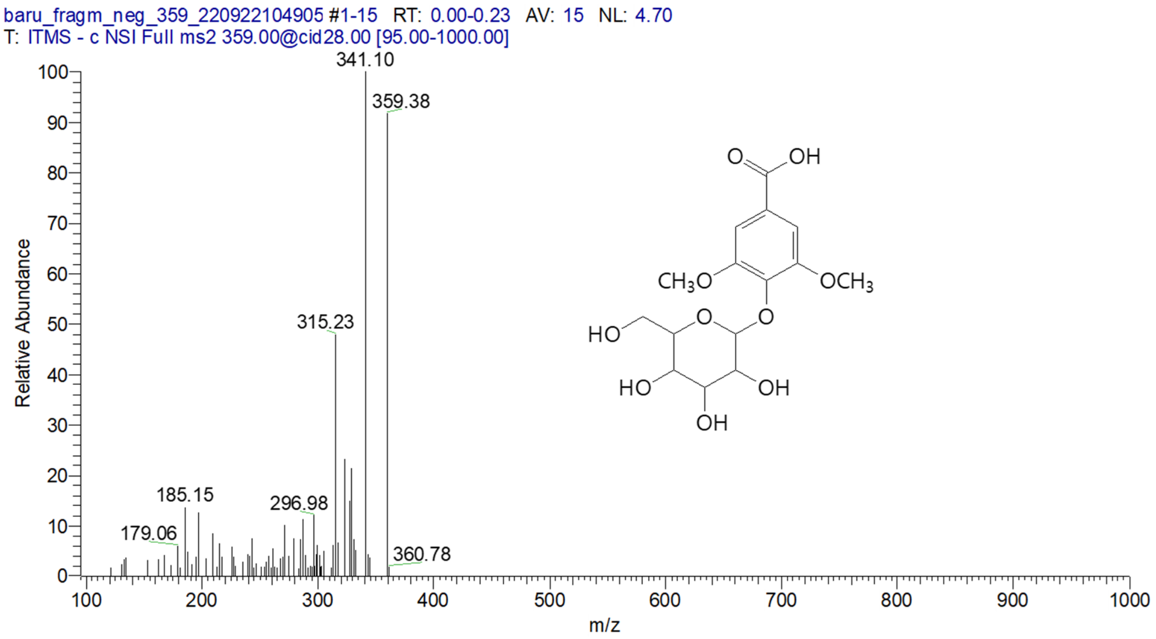

Figure S22. Product ion mass spectrum (MS/MS) of the ion of m/z 431 (ascribed as deprotonated Isovitexin).

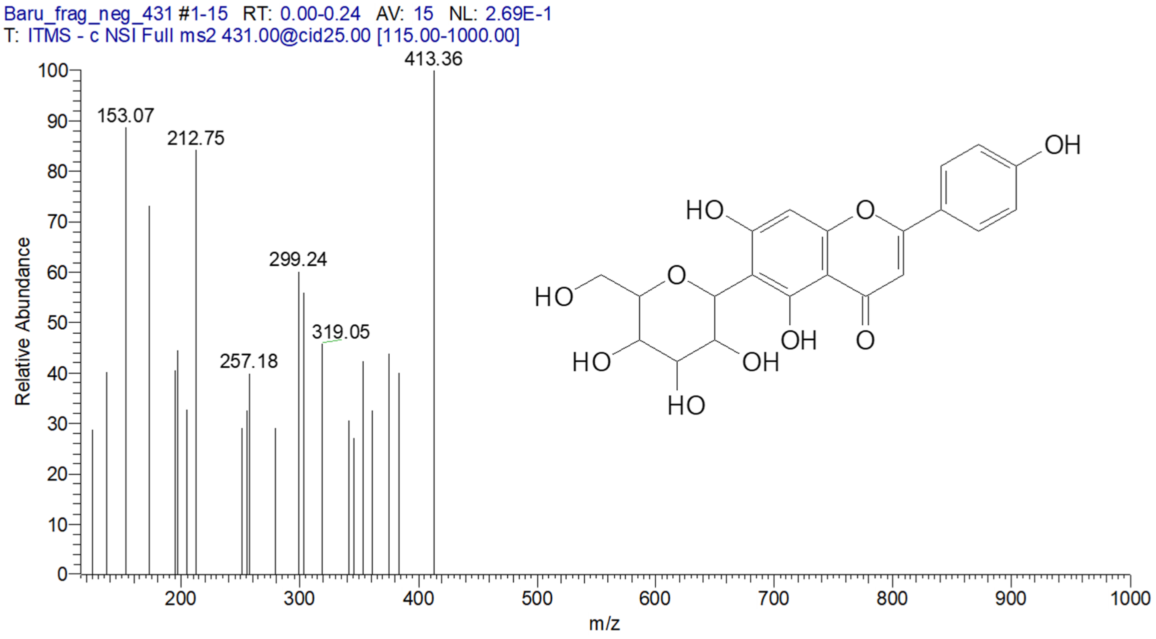

Figure S23. Product ion mass spectrum (MS/MS) of the ion of m/z 449 (ascribed as deprotonated Eriodictyol-O-glucoside).

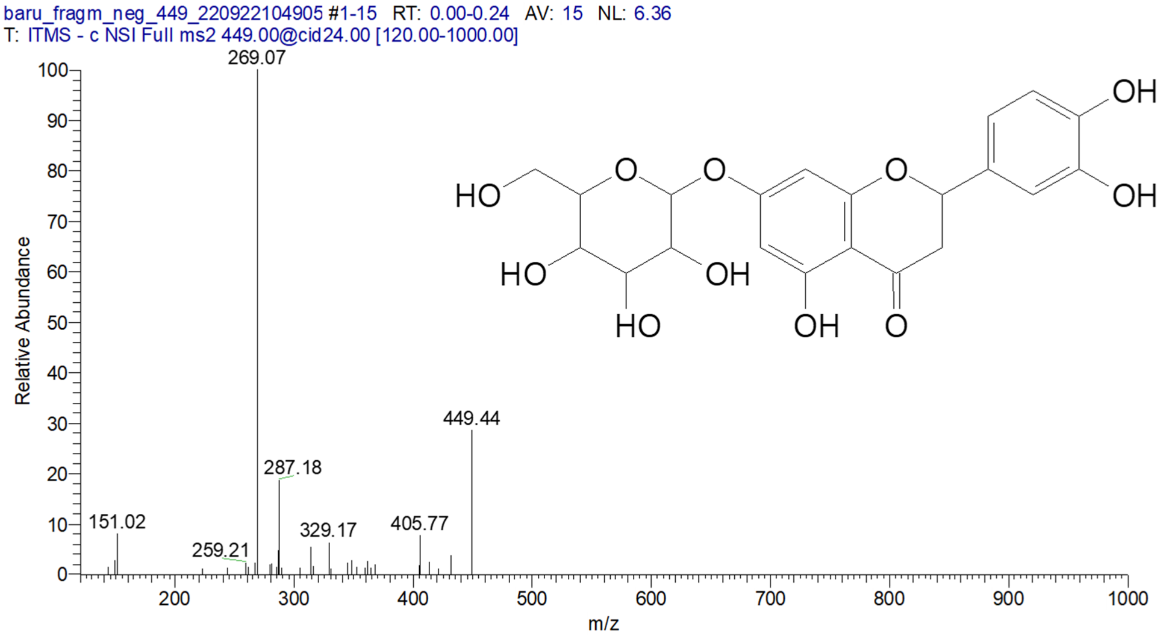

Figure S24. Product ion mass spectrum (MS/MS) of the ion of m/z 477 (ascribed as deprotonated Quercetin-3-O-glucouronide).

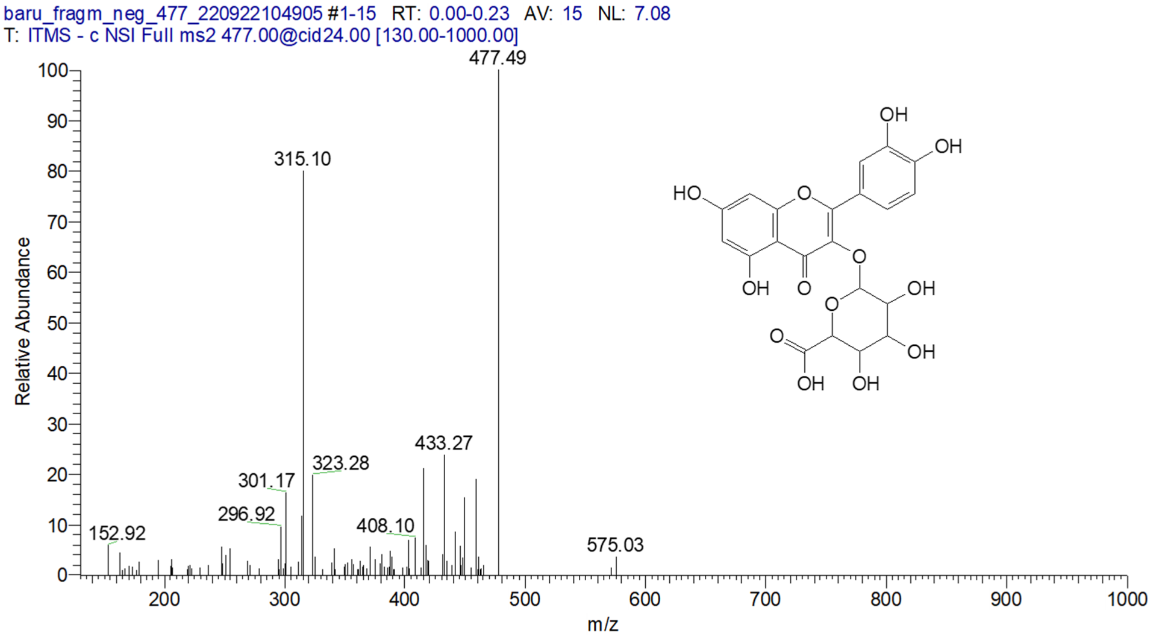

Figure S25. Product ion mass spectrum (MS/MS) of the ion of m/z 481 (ascribed as deprotonated Dihydromyricetin (ampelopsin) 3′-O-β-D-glucopyranoside).

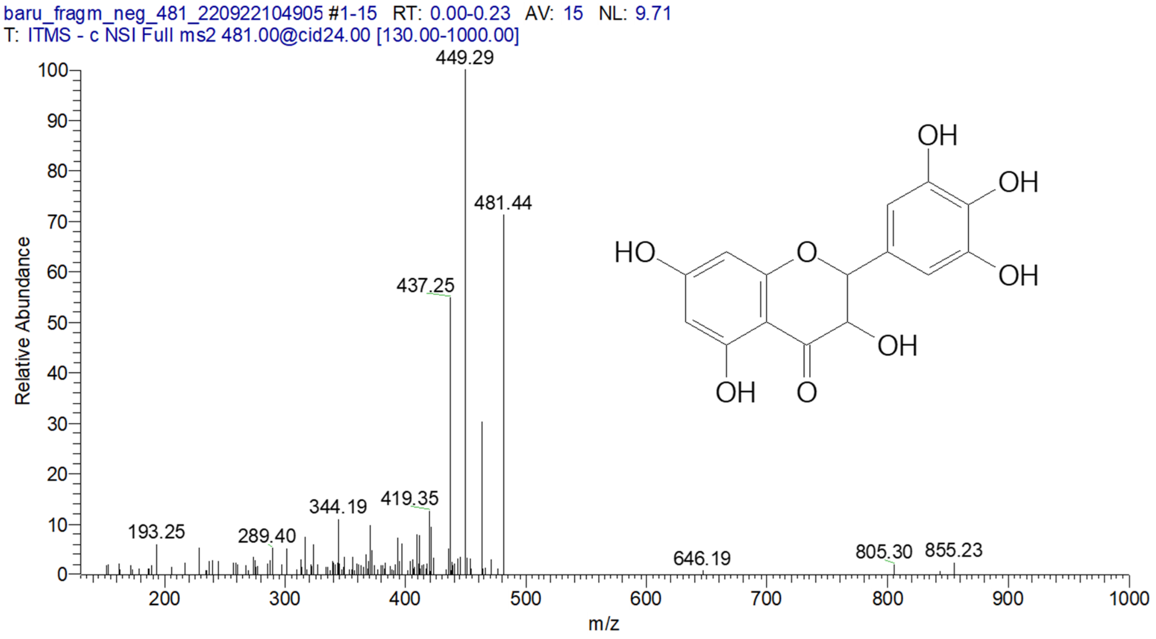

Figure S26. Product ion mass spectrum (MS/MS) of the ion of m/z 533 (ascribed as deprotonated Noricaritin hexoside).

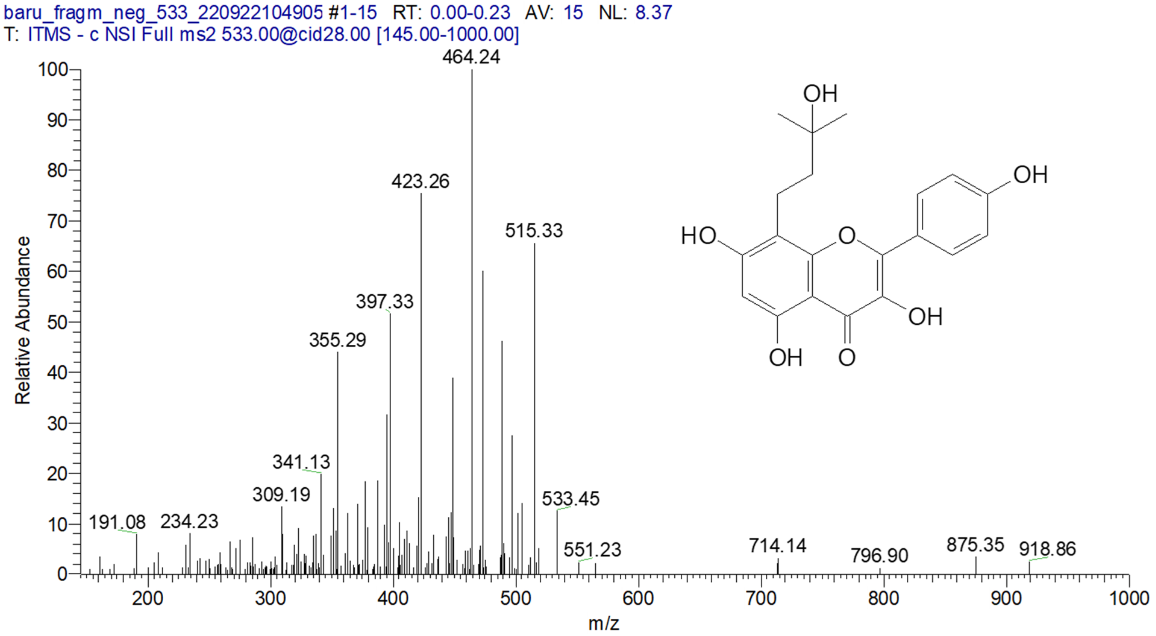

Figure S27. Product ion mass spectrum (MS/MS) of the ion of m/z 567 (ascribed as deprotonated Phloretinxylo glucoside).

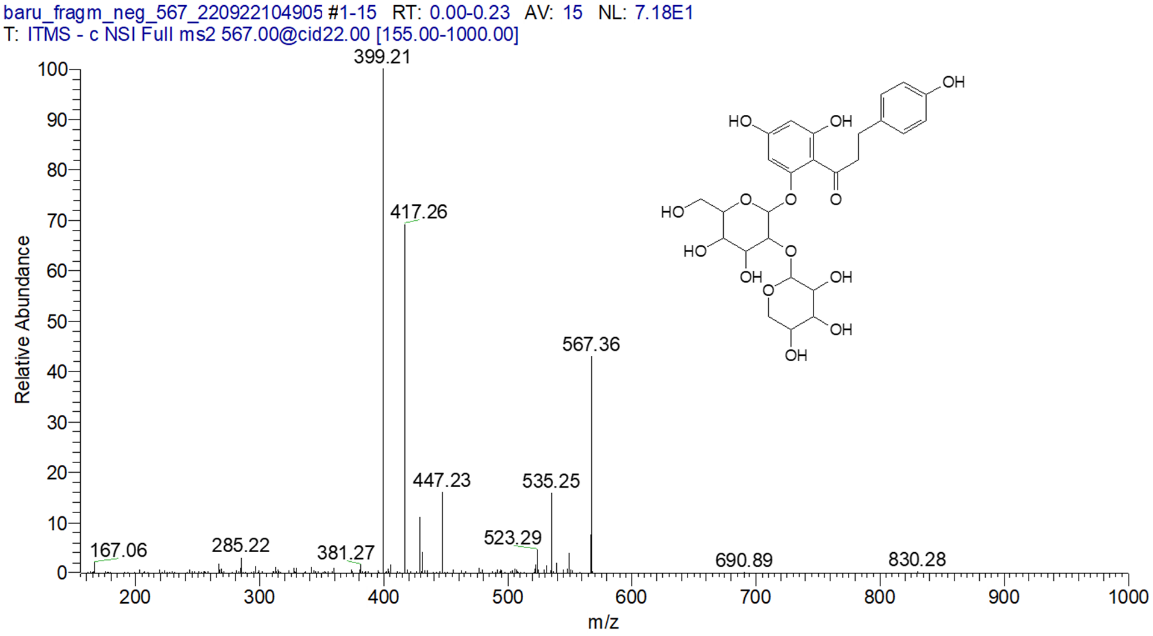

Figure S28. Product ion mass spectrum (MS/MS) of the ion of m/z 597 (ascribed as deprotonated Phloretin-3',5'-di-C-glucoside).

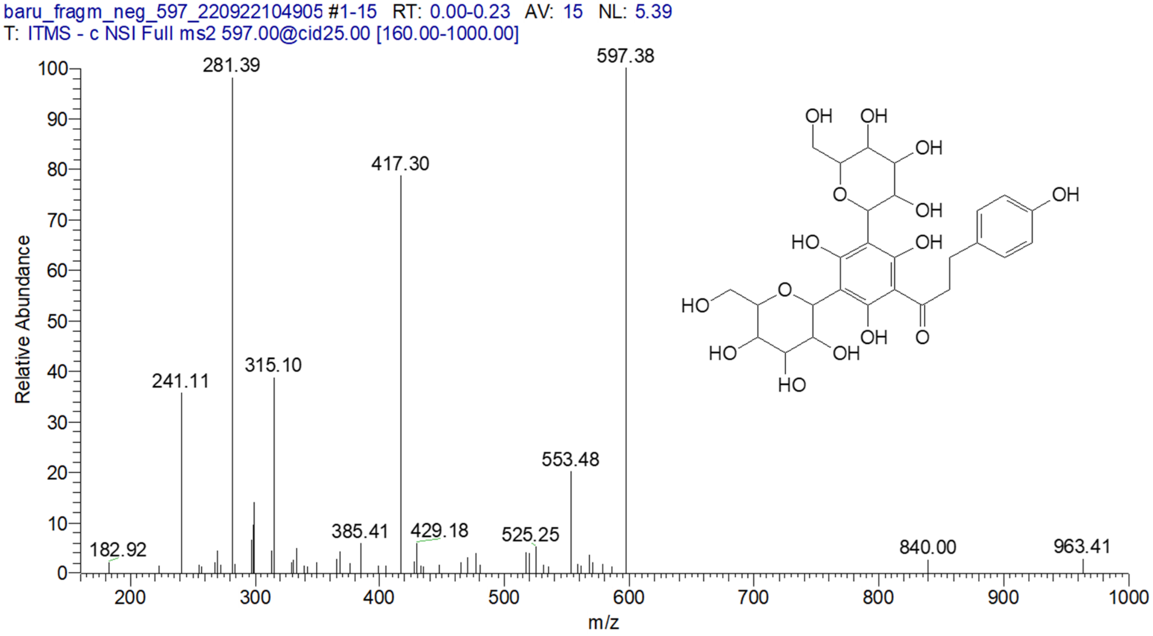

Figure S29. Product ion mass spectrum (MS/MS) of the ion of m/z 623 (ascribed as deprotonated Isorhamnetin3-O-(2''-α-arabinopyranosyl)-β-glucopyranoside).

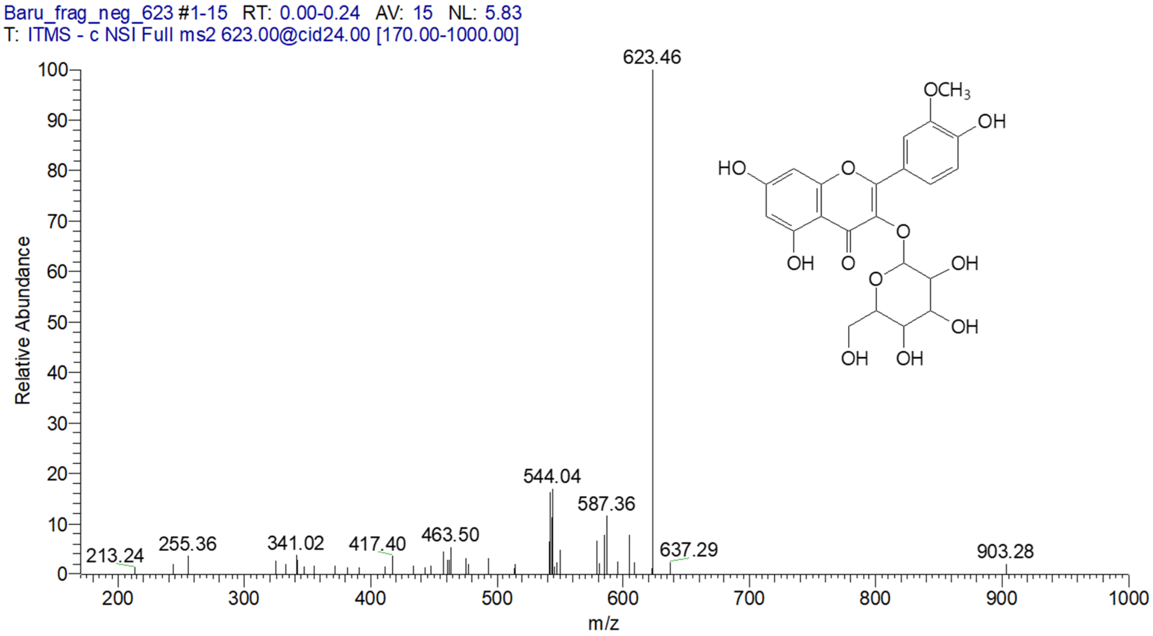

Figure S30. Product ion mass spectrum (MS/MS) of the ion of m/z 850 (ascribed as deprotonated Procyanidin trimer).

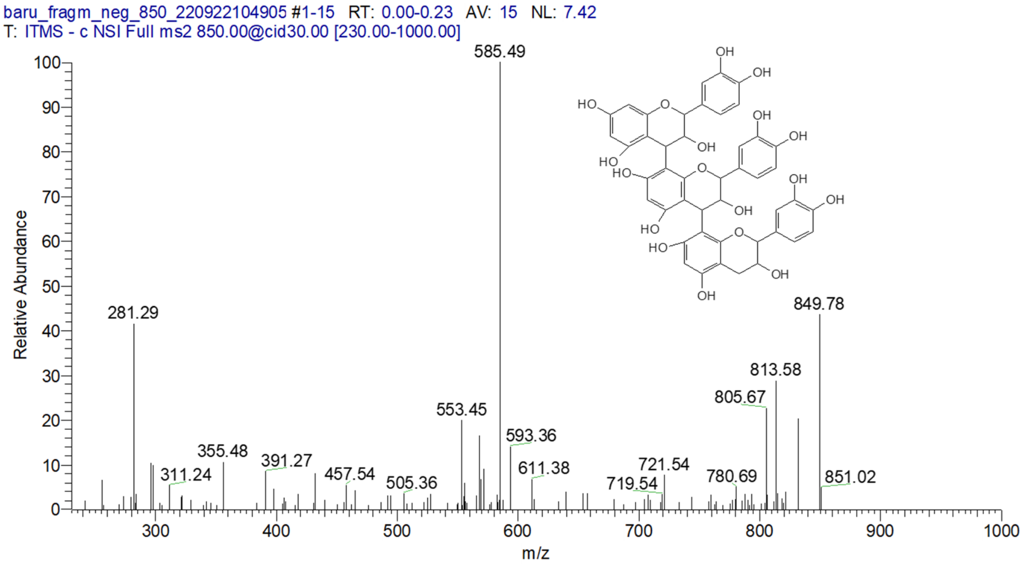

Figure S31. Product ion mass spectrum (MS/MS) of the ion of m/z 285 (ascribed as protonated Calycosin).

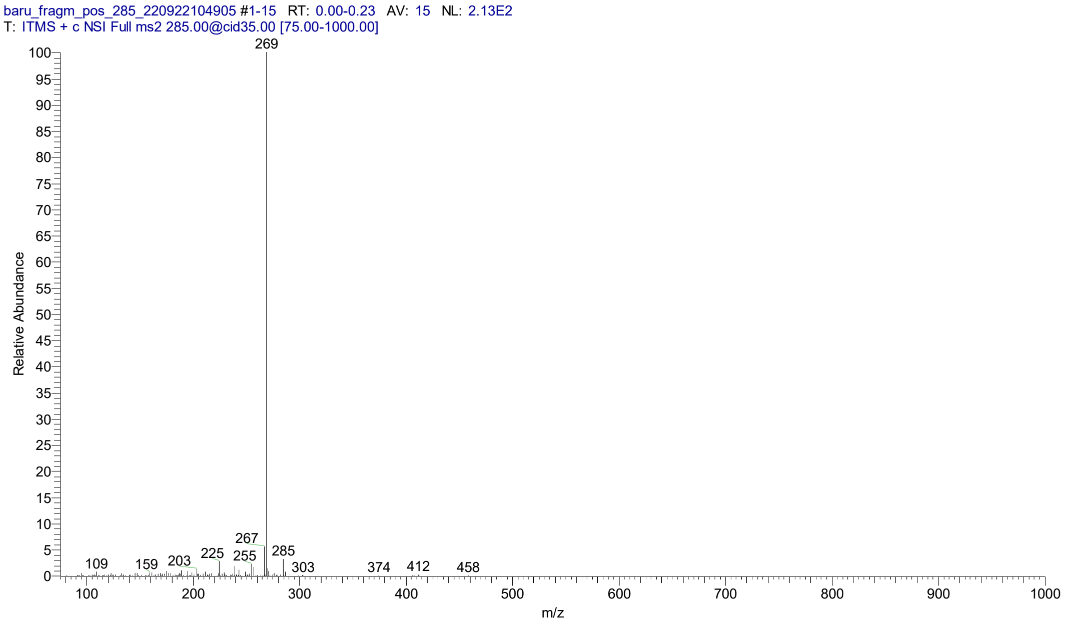

Figure S32. Product ion mass spectrum (MS/MS) of the ion of m/z 317 (ascribed as protonated 4,10-Dihydroxy-3,9-dimethoxypterocarpan).

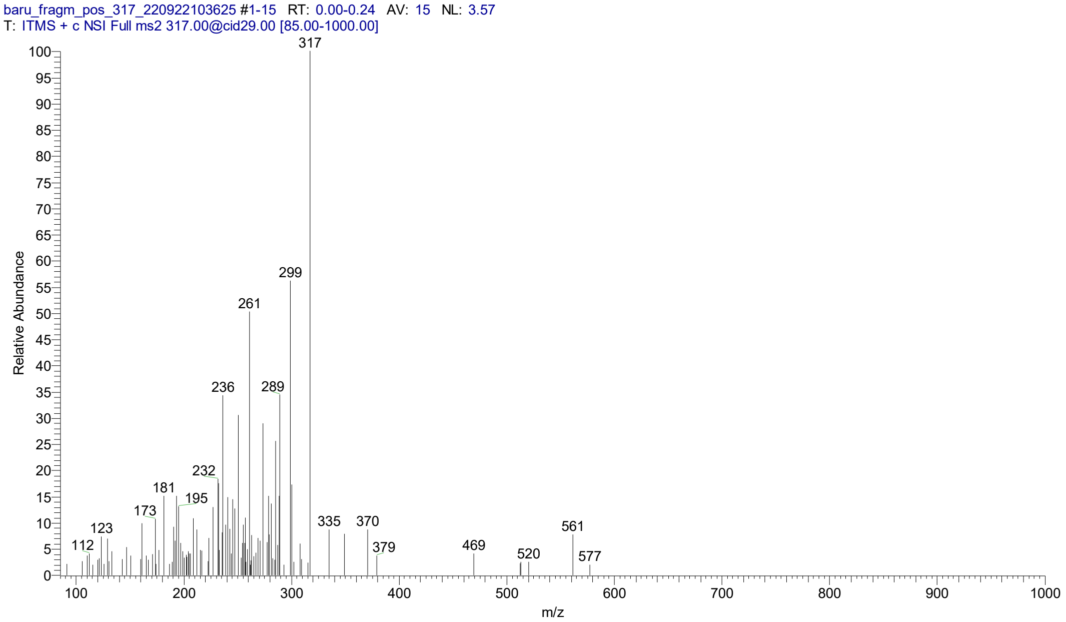

Figure S33. Product ion mass spectrum (MS/MS) of the ion of m/z 395 (ascribed as protonated Stigmasterol).

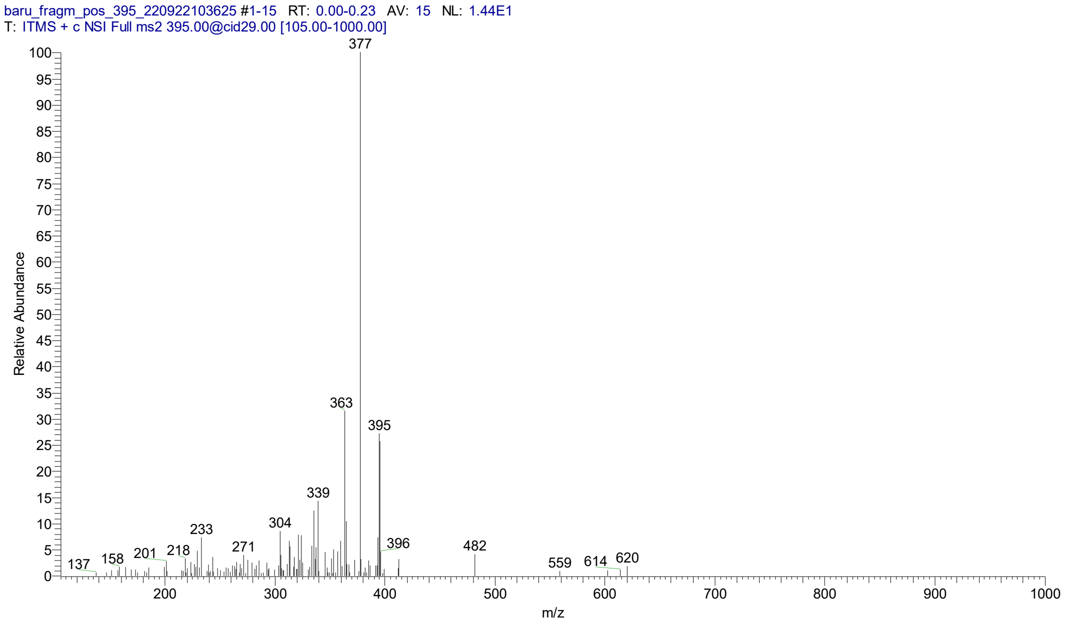

Figure S34. Product ion mass spectrum (MS/MS) of the ion of m/z 678 (ascribed as protonated Tricaffeoyl-quinic acid).

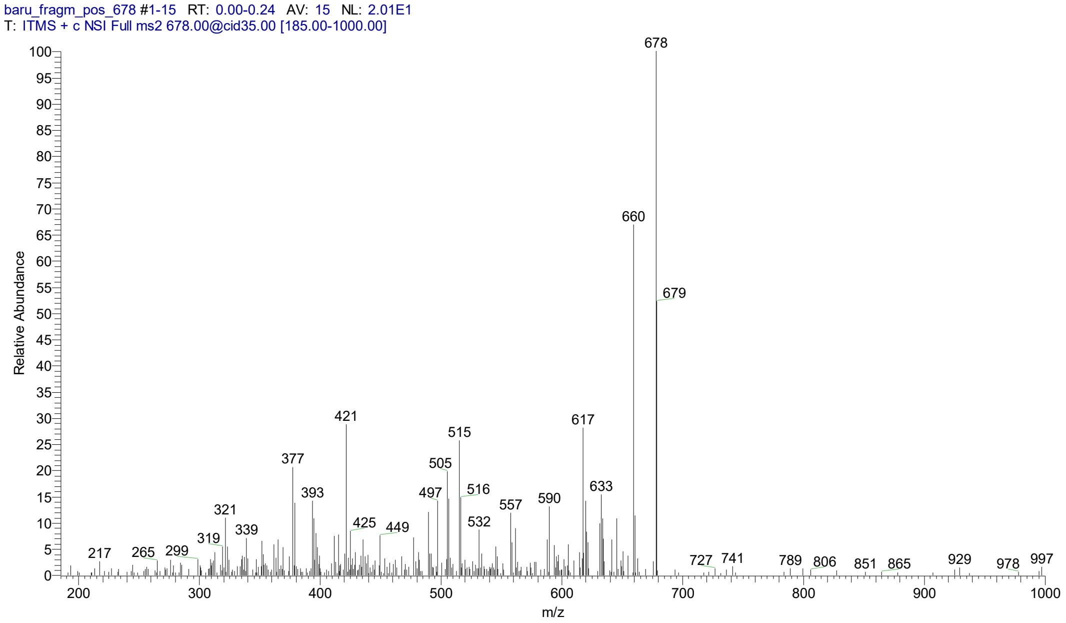

Figure S35. Product ion mass spectrum (MS/MS) of the ion of m/z 741 (ascribed as protonated Sutherlandin).

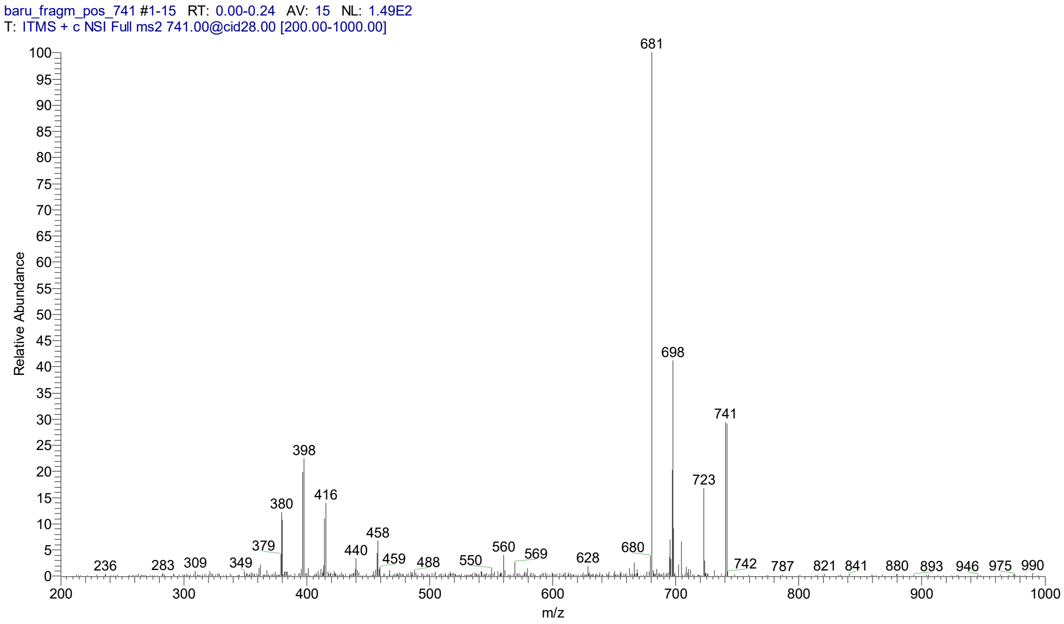

Figure S36. Product ion mass spectrum (MS/MS) of the ion of m/z 799 (ascribed as protonated Ellagic acid derivative).

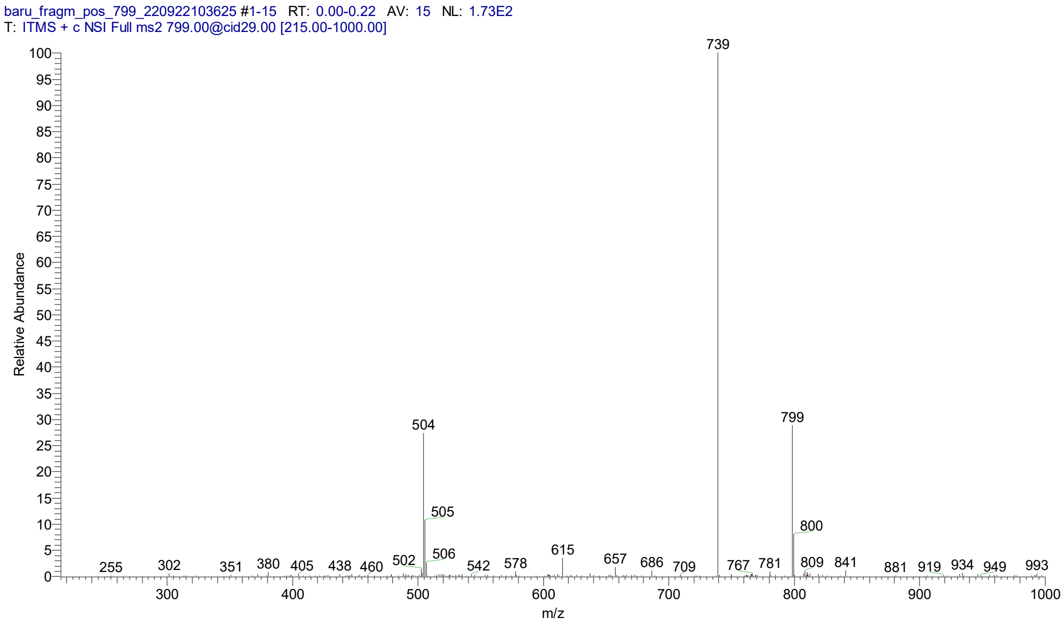

Figure S37. Product ion mass spectrum (MS/MS) of the ion of m/z 841 (ascribed as protonated Schoepfin A derivative of (iso)mangiferin).

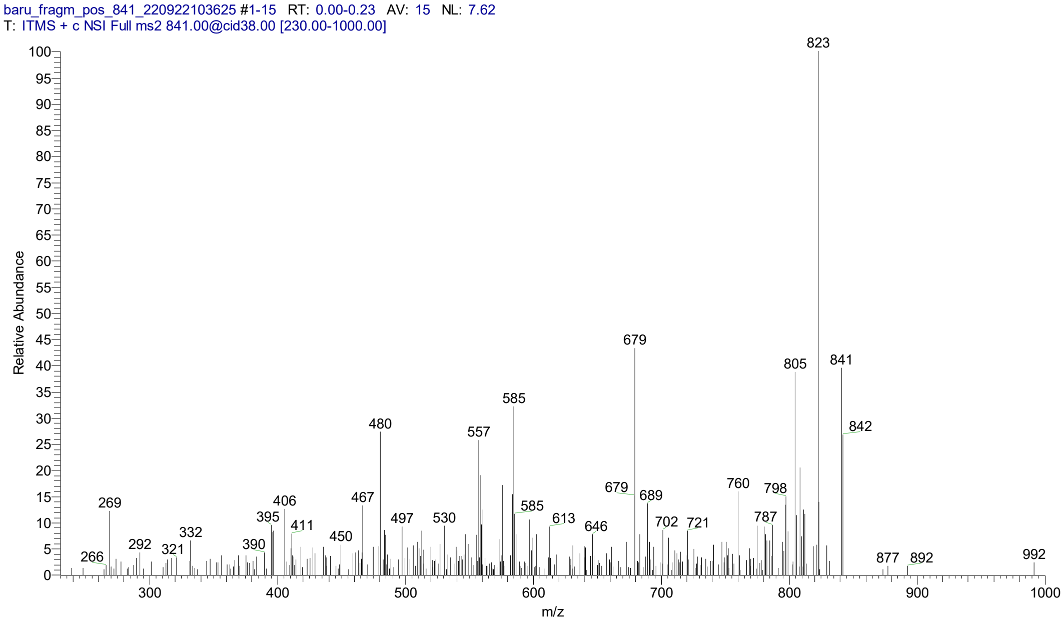

Figure S38. Product ion mass spectrum (MS/MS) of the ion of m/z 857 (ascribed as protonated Nothofagin derivative of (iso)mangiferin).

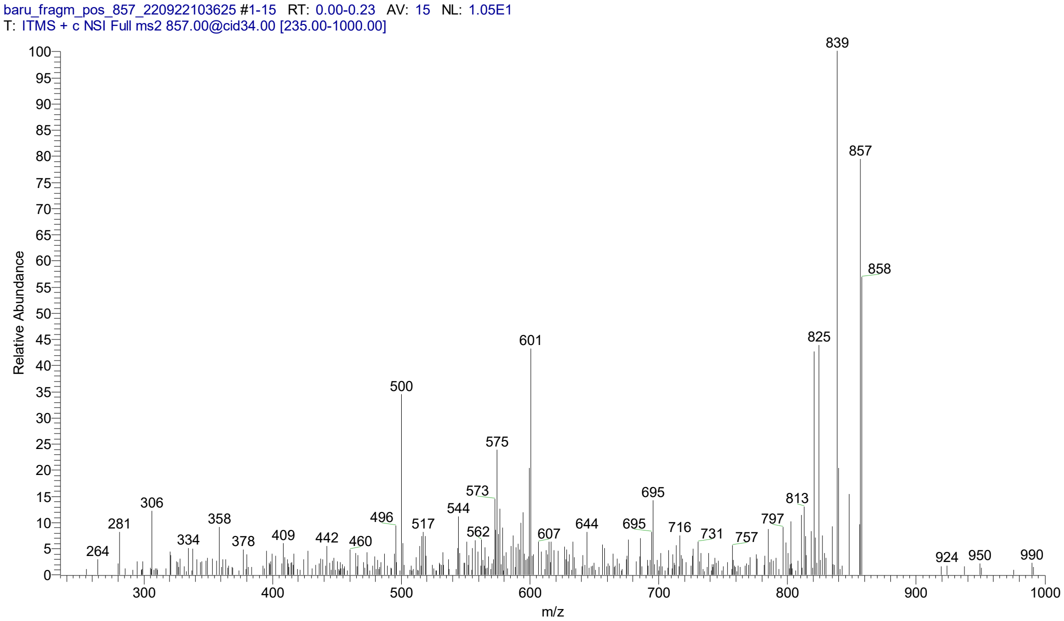

Figure S39. Product ion mass spectrum (MS/MS) of the ion of m/z 870 (ascribed as protonated Astragaloside I).

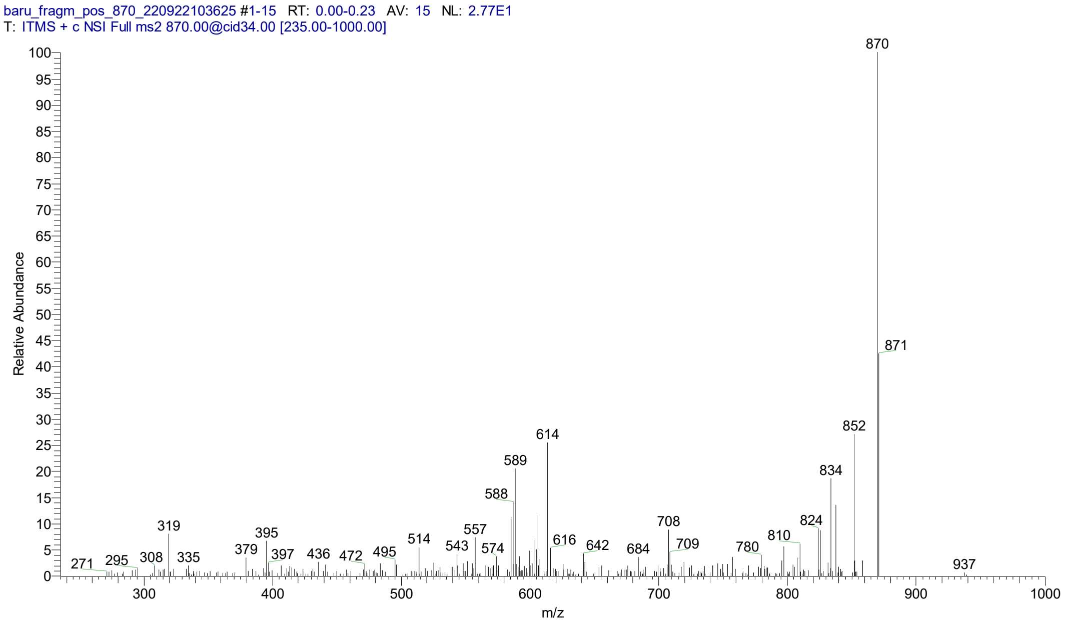

Figure S40. Product ion mass spectrum (MS/MS) of the ion of m/z 873 (ascribed as protonated Aspalathin derivative of (iso)mangiferin).

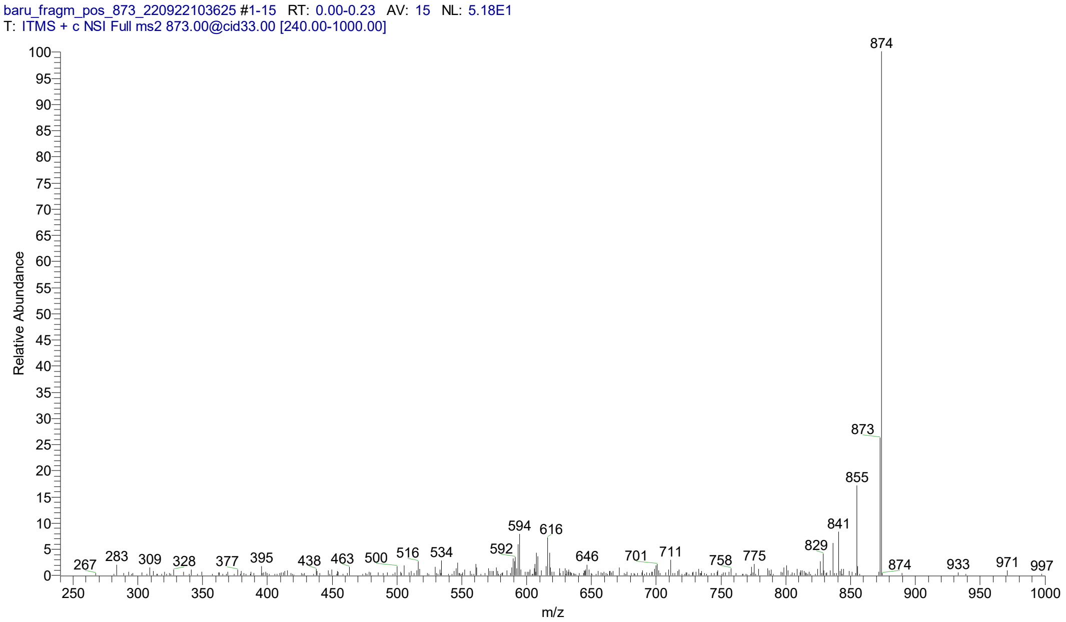

Supplement: Supplementary file 1 [file plants-13-01833-s001.zip › plants-2819708-supplementary.pdf]
